# Socioeconomic differentiation in sport engagement: frequent exercise, live sport attendance, and their overlap in the 2023 Chinese General Social Survey

**DOI:** 10.3389/fpubh.2026.1965653

**Published:** 2026-10-07

**Authors:** Congfei Lu, Huili Dai, Jianhua Xu

**Affiliations:** 1 School of Physical Education and Sport Science, Fujian Normal University, Fuzhou, Fujian, China; 2 Fujian Provincial Heavy Athletic Sports Management Center, Fuzhou, Fujian, China

**Keywords:** Chinese General Social Survey, exercise, live sport attendance, multinomial logistic regression, socioeconomic position, urban–rural residence

## Abstract

**Objective:**

To examine how education, personal income, subjective social status, and urban–rural residence were associated with joint categories of frequent exercise and live sport attendance among adult respondents in CGSS2023.

**Methods:**

This cross-sectional study included 5,585 adults from the 2023 Chinese General Social Survey. Frequent leisure-time exercise and any past-year live attendance were cross-classified into low, active-only, spectator-only, and dual engagement. Weighted percentages described the category distribution, and covariate-adjusted associations were estimated using an unweighted multinomial logistic model with robust standard errors. Sensitivity analyses varied weighting, sample composition and outcome definitions. Exploratory interaction tests were adjusted for multiple comparisons.

**Results:**

Education showed the most consistent associations, with the largest relative risk ratio for dual engagement in the primary unweighted model (1.185, 95% CI 1.148–1.224). Urban residence was also positively associated with dual engagement in the primary unweighted model (relative risk ratio 2.287, 95% CI 1.753–2.985). The corresponding estimates were 1.412 and 1.458 in the combined-weight and selection-adjusted models, respectively; none of the three urban–rural contrasts was statistically significant in either model. Income associations varied with income coding, work-status adjustment, weighting and the attendance threshold. Associations with subjective social status were modest. The education-by-residence interaction was statistically significant after multiplicity adjustment in the unweighted linear-score model. The categorical interaction was not statistically significant under combined weighting.

**Conclusion:**

In the CGSS2023 analytical sample, socioeconomic patterning differed across combinations of frequent leisure-time exercise and live sport attendance. Education was positively associated with active-only and dual engagement across the weighting specifications examined, while income associations varied with model specification and evidence for urban–rural differences depended on weighting. Monitoring sport engagement should distinguish exercise from live attendance and examine their overlap to identify the forms of engagement in which socioeconomic differences are concentrated.

## 1 Introduction

Exercise and attendance at live sporting events are distinct forms of sport engagement. Exercise contributes directly to physical activity, and insufficient physical activity remains a major global public-health concern (1, 2). Live attendance is a form of leisure and social participation associated with subjective wellbeing and social connectedness (3, 4). Studying the two behaviors jointly distinguishes active participation, spectator involvement and their overlap.

Exercise and live attendance are usually analyzed separately, including in studies based on Chinese national surveys (5–10). Separate outcomes cannot show whether socioeconomic differences are concentrated among people who exercise frequently without attending, attend without frequent exercise, or combine the two. This gap is particularly relevant in China, where expanded institutional support for mass participation has coexisted with persistent educational, economic and urban–rural differences in sport involvement (5–8, 11).

Using data from the 2023 Chinese General Social Survey (CGSS2023) (12), we classified respondents into four mutually exclusive joint sport-engagement categories: low engagement, active-only engagement, spectator-only engagement and dual engagement. We described their distribution and examined covariate-adjusted associations with education, personal income, subjective social status and urban–rural residence in the analytical sample. Exploratory analyses examined whether these associations varied by sex, age and residence. The joint categories allow these associations to be located within specific combinations of exercise and attendance.

## 2 Literature review, theoretical framework and hypotheses

Exercise and live sport attendance have largely been studied in separate research traditions. Exercise is commonly studied as physical activity or health behavior, whereas attendance is more often approached as leisure consumption, cultural practice or spectatorship. Comparative research identifies both shared and distinct correlates of the two behaviors (9). A binary measure of frequent exercise groups active-only with dual engagement; a binary attendance measure groups spectator-only with dual engagement. Cross-classification distinguishes the socioeconomic associations within these combinations.

Once the two behaviors are considered jointly, socioeconomic patterning need not follow a single status gradient. Education and income represent different dimensions of social position and may relate differently to the same behavior (13, 14). Bourdieu's work on sport likewise emphasizes that practices depend on the composition of cultural and economic resources, not simply their total amount (15, 16). Research on subjective social status adds perceived relative position, while leisure-constraints research directs attention beyond individual resources to time, cost, transport and the availability of opportunities (17, 18). Together, these literatures support a distinction among educational resources, material resources, perceived social position and residential context, represented here by education, personal income, subjective social status and urban–rural residence.

Educational attainment may shape sport engagement through knowledge, socialization and familiarity with organized activity. Chinese and international studies generally report greater exercise or sport participation among more educated adults, although gradients vary across population groups and types of sport (5–7, 19). Education has also been associated with event watching and live sport attendance (6, 9, 20, 21), but the larger body of evidence concerns active participation. This literature provides the basis for linking education primarily to joint categories that include frequent exercise.

Income represents current material capacity rather than educational or cultural resources. Its relationship with active sport participation varies across activities, whose financial requirements differ substantially (5, 6, 19). Live attendance can involve ticket, travel and other discretionary costs, and comparative research reports income differences in attendance at sporting events (9, 21, 22). Income is therefore expected to differentiate joint categories that include live attendance.

Subjective social status captures how individuals locate themselves within the social hierarchy and is related to, but not reducible to, objective socioeconomic indicators (17). Evidence from five CGSS waves shows that higher subjective social status is associated with greater physical activity after education, income and other characteristics are considered (8). With substantially less evidence available for live attendance, the directional prediction concerns categories that include frequent exercise.

Urban–rural residence shifts the analysis from personal position to the setting in which sport engagement occurs. Residential environments differ in transport, facilities, organized activities and access to sporting events (18, 23, 24). Chinese survey studies also report urban–rural differences in exercise and event watching (5, 6). Because attending a live event depends directly on its location, timing and accessibility, the directional prediction concerns categories that include attendance.

With low engagement as the reference category, we proposed the following hypotheses:

**H1**: higher educational attainment is expected to be associated with a greater relative likelihood of active-only and dual engagement.

**H2**: higher personal income is expected to be associated with a greater relative likelihood of spectator-only and dual engagement.

**H3**: higher subjective social status is expected to be associated with a greater relative likelihood of active-only and dual engagement.

**H4**: urban residence is expected to be associated with a greater relative likelihood of spectator-only and dual engagement.

## 3 Materials and methods

### 3.1 Data source and study design

Data came from CGSS2023, a nationwide cross-sectional survey of Chinese residents (12). The released file contains 11,326 interviews: 5,583 face-to-face and 5,743 by telephone. Reporting followed the STROBE recommendations for cross-sectional studies (25).

### 3.2 Analytical sample and sample selection

Of the 11,242 respondents aged 18–100 years, 5,908 had valid responses to both the physical exercise and live sport attendance items. These comprised 5,551 of 5,583 face-to-face respondents (99.4%) and 357 of 5,659 telephone respondents (6.3%). The telephone questionnaire specified randomized administration of the leisure-activity subitems. We subsequently excluded 323 respondents (5.5%): 313 for missing focal socioeconomic indicators and a further 10 for missing pre-specified covariates. The final complete-case sample comprised 5,585 adults, including 5,261 face-to-face respondents (94.2%) and 324 telephone respondents (5.8%) (Figure 1; Supplementary Table S2). Supplementary Table S3 compares included and excluded respondents with valid joint-outcome data (Panel A) and age-eligible telephone respondents with both sport items valid vs. at least one item missing (Panel B).

**Figure 1:**
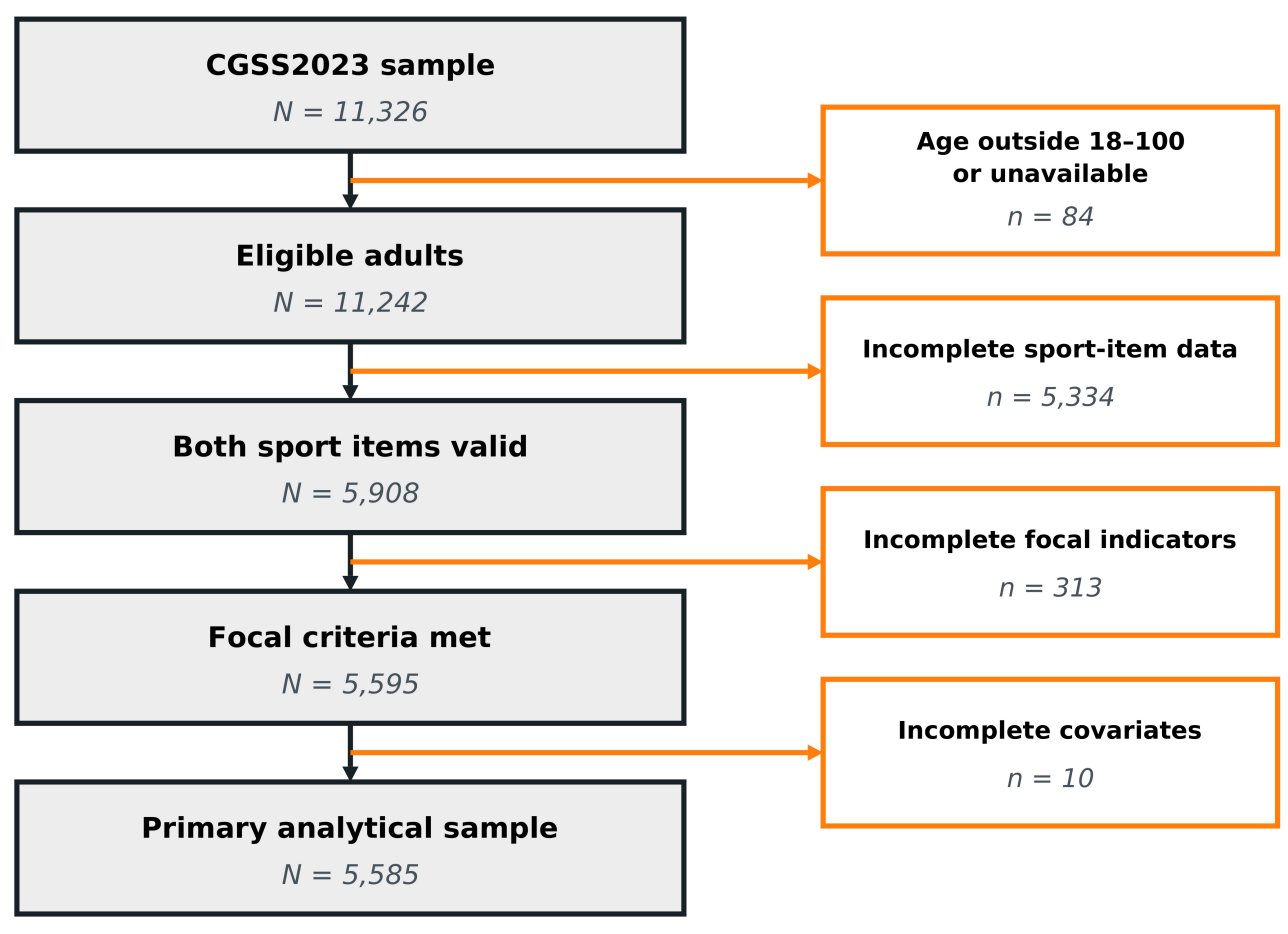
Derivation of the primary analytical sample.

### 3.3 Construction of the joint sport-engagement categories

Respondents reported how often they exercised (a3009) and attended sporting events in person (a3010) during their free time over the past year. Both items used five response categories: every day, several times a week, several times a month, several times a year or less, and never. Non-substantive responses were coded missing.

Exercise every day or several times a week was coded as frequent; lower frequencies were not. Any live attendance during the year was coded as attendance, and never as no attendance. The thresholds capture frequent exercise alongside any involvement in live spectatorship. Crossing the indicators produced four nominal joint categories: low engagement (no attendance and exercise below the frequent threshold), active-only (frequent exercise without attendance), spectator-only (live attendance without frequent exercise) and dual engagement (frequent exercise with any past-year attendance). The low and spectator-only categories include occasional exercisers; dual engagement does not require frequent attendance.

### 3.4 Socioeconomic indicators

The model included three individual-level indicators (education, personal income and subjective social status) plus residential context.

#### 3.4.1 Educational attainment

Educational attainment came from the highest reported education level (a7a) and was converted to years-equivalent values: 0 for no schooling or literacy classes, 6 for primary, 9 for junior middle, 12 for upper-secondary or vocational, 15 for college, 16 for bachelor's and 19 for postgraduate education. These values represent levels, not observed years completed. The primary multinomial model treated this score as linear, assuming a constant change in each category-vs.-reference log probability ratio per score unit. Categorical models and tests of this restriction are reported in Supplementary Table S7, Panels A1–A4.

#### 3.4.2 Personal income

Personal income was measured as total personal income in 2022, in yuan (a8a). Zero and all observed positive amounts, including values above 1,000,000 yuan, were retained. The CGSS top-code, negative values and non-substantive responses were coded missing. Because the retained distribution was strongly right-skewed, income entered the primary model as ln (income + 1), with one yuan added to retain zero-income observations.

#### 3.4.3 Subjective social status

Subjective social status came from a43a, a ladder from 1 (lowest) to 10 (highest), and was entered as a linear score.

#### 3.4.4 Residential context

Residential context came from a25a. City centers, urban fringes or transition zones, and towns were coded urban (1); villages were coded rural (0).

### 3.5 Covariates

Covariates in the primary model were age, age squared, sex, self-rated health, household registration (hukou), partnered status, survey mode and province. Age was calculated from birth year and centered at 50 before squaring. Self-rated health ranged from 1 (very unhealthy) to 5 (very healthy). Agricultural hukou and resident hukou formerly classified as agricultural were combined; non-agricultural hukou and resident hukou formerly classified as non-agricultural formed the reference group. Cohabiting respondents and those in a first marriage or remarriage with a spouse were coded as partnered. Province was represented by indicators. Supplementary Table S1 gives source items and coding.

### 3.6 Statistical analysis

The joint sport-engagement categories and analytical-sample characteristics were summarized using unweighted counts and descriptive statistics. For weighted prevalence estimates, we multiplied the CGSS integrated survey weight (weight2) by the inverse proportion of age-eligible respondents with valid responses to both sport items within each interview mode. The resulting weights were normalized to a mean of one in the complete-case sample (Supplementary Table S5). This adjustment assumes that, after survey weighting, respondents with both items valid represent the joint-outcome distribution within each mode. The primary regression targeted conditional associations in the complete-case sample, using an unweighted multinomial model with survey mode and province indicators (26, 27). We compared the direction, magnitude and uncertainty of the unweighted and weighted estimates.

#### 3.6.1 Primary multinomial regression model

Because the four joint sport-engagement categories were nominal and unordered, multinomial logistic regression was fitted with low engagement as the reference category (28). Education, log-transformed income, subjective social status and urban residence were entered simultaneously, adjusting for centered age, age squared, sex, self-rated health, hukou, partnered status, survey mode and province indicators. To address sparse province-by-category cells, we pooled the 12 provinces with fewer than 20 complete cases each into a single adjustment category (Supplementary Table S9, Panel E). For category *k* in {active-only, spectator-only, dual engagement}, with low engagement coded as *Y*_*i*_ = 0, the model was:

$$\begin{array}{l} log\left[\frac{Pr\left(Y_{i}=k\right)}{Pr\left(Y_{i}=0\right)}\right]=\alpha_{k}+\beta_{k}^{T}S_{i}+\gamma_{k}^{T}X_{i}+\delta_{p\left(i\right),k}+\theta_{k}Mode_{i} \end{array}$$

Here, S_*i*_ contains education, log-transformed personal income, subjective social status, and urban residence; X_*i*_ contains centered age, age squared, sex, self-rated health, hukou, and partnered status. δ_*p*(*i*), *k*_ denotes the category-specific province fixed effect for respondent *i*, and Mode_*i*_ denotes the survey-mode indicator. We used respondent-level robust sandwich standard errors without small-sample correction for the primary unweighted and combined-weight models. Weights were treated as fixed when calculating standard errors for the weighted model. Results are reported as relative risk ratios (RRRs), adjusted probabilities, and average marginal effects with 95% confidence intervals. Education contrasts represent one-unit increases in the assigned years-equivalent score, subjective social status contrasts represent one-rung increases, and residence compares urban with rural respondents. Income RRRs represent a doubling of (income + 1), equivalent to a ln(2)-unit increase in ln (income + 1). To express income differences in yuan, we also report adjusted probability contrasts for a change in annual income from 3,000 to 50,000 yuan, the analytical sample's first and third quartiles.

#### 3.6.2 Robustness analyses

Robustness analyses varied weighting, outcome definitions, variable coding and covariate adjustment. Models were re-estimated using the combined survey/item-observation weight and the face-to-face household weight. For the selection-adjusted model, we divided the combined weight by the estimated probability of complete-case inclusion (29). The inclusion model is specified in Supplementary Table S5, Panel A. All analysis weights were normalized to mean one. Only the selection-adjusted weights were trimmed by capping them at the 1st and 99th percentiles, then renormalized to mean one. We also fitted an unweighted model in the 5,261 face-to-face complete cases, retaining the primary covariates except survey mode (Supplementary Table S8, Panel B). Outcome sensitivity analyses used the same 5,585 complete cases and were fitted unweighted and with combined weights. We modeled the original five-category items separately, raised the attendance threshold to at least several times a month, and split low engagement into never vs. occasional exercise, both without attendance. The split-reference model used never exercise and never attendance as the reference and retained the other three categories. Their adjusted probability contrasts were compared with a four-category model using the same adjustment set (Supplementary Table S6).

Province indicators were omitted from the stricter-attendance, split-reference and original attendance-frequency models because of separation; the exercise-frequency model and four-category comparator used matching adjustment sets. Separate binary models retained province indicators. These outcome sensitivity analyses used respondent-level robust inference, treating weights as fixed, without multiplicity adjustment. To assess the education-score assumption, we replaced the linear term with indicators for the seven attainment categories, keeping the sample and covariates unchanged. In unweighted and combined-weight models, robust joint Wald tests evaluated the 15 linear-score restrictions across the three non-reference equations. Predicted probabilities were averaged equally over the same 5,585 covariate profiles; we also compared dual-engagement probabilities between postgraduate and bachelor's education (Supplementary Table S7, Panels A1–A4). Other checks varied the coding of subjective social status and residence, excluded incomes above 1,000,000 yuan, and omitted self-rated health, province indicators or hukou.

To examine zero incomes, we replaced ln (income + 1) with two jointly entered terms: a zero-income indicator and a separate term equal to ln (income/20,000) for positive incomes and zero otherwise. The indicator compared zero income with 20,000 yuan, the sample median; the positive-income term described variation among respondents with positive incomes. These models used the same 5,585 respondents and were fitted unweighted and with combined weights (Supplementary Table S7, Panels G–H).

Work-status analyses used 5,256 face-to-face respondents with classifiable responses to items a53 and a54, distinguishing working or temporarily absent from work (reference), retired and not working, and otherwise not working. These items were not administered in telephone interviews. For each income specification, models with and without these indicators used identical respondents and were fitted unweighted and with household weights. Survey mode was omitted because these analyses included only face-to-face respondents (Supplementary Table S8, Panel C).

#### 3.6.3 Exploratory heterogeneity analysis

Exploratory heterogeneity was assessed in extensions of the primary model by interacting education, log-transformed income and subjective social status with sex, age group (18–59 vs. ≥60 years) and residence, yielding nine interaction tests. Each interaction was evaluated using an omnibus Wald test across the three non-reference equations. The Benjamini–Hochberg procedure controlled the false discovery rate across the nine tests (30), with interaction results evaluated using adjusted q values. For interactions with *q* < 0.05, subgroup-specific associations were summarized using adjusted predicted probabilities and category-specific relative risk ratios.

To assess education coding in the residence interaction, we fitted categorical-education models under both weighting specifications. The unrestricted seven-level model showed separation because the seven rural postgraduate respondents included no low-engagement cases. We therefore pooled bachelor's and postgraduate education for this interaction check, retaining all 5,585 cases. Robust Wald tests assessed the interaction and the linear-score restrictions using matching six-level coding. Predictions were standardized equally over the same covariate profiles, and urban–rural probability differences were calculated at each education level. These additional checks were outside the original nine-test family and used unadjusted *p*-values (Supplementary Table S9, Panels G1–G3 and H1–H3).

## 4 Results

### 4.1 Sample selection, analytical-sample characteristics and category prevalence

The analytical sample had a mean age of 53.94 years (SD 16.44), and just over half were female (54.0%) or lived in urban areas (54.5%); 68.5% had an agricultural or formerly agricultural hukou, and median annual personal income was 20,000 yuan (IQR 3,000–50,000). Zero income was reported by 932 respondents (16.7%; 14.9% with combined weights). Low engagement was the largest category in the unweighted sample, comprising 2,992 respondents (53.6%), followed by active-only engagement (1,326; 23.7%), spectator-only engagement (637; 11.4%) and dual engagement (630; 11.3%). After combined weighting, the estimated proportions were 41.3%, 18.4%, 18.4% and 21.9%, respectively (Table 1).

**Table 1:** Analytical-sample characteristics and estimated joint sport-engagement category prevalence.

Panel A. Prevalence of the joint sport-engagement categories		
Category	Unweighted n (%)	Weighted prevalence (%)
Low engagement	2,992 (53.6%)	41.3%
Active-only engagement	1,326 (23.7%)	18.4%
Spectator-only engagement	637 (11.4%)	18.4%
Dual engagement	630 (11.3%)	21.9%
Panel B. Characteristics of the analytical sample		
Characteristic	Unweighted estimate	
Age, years, mean (SD)	53.94 (16.44)	
Education, years-equivalent, mean (SD)	9.31 (4.64)	
Annual personal income, yuan, median [IQR]	20,000 [3,000–50,000]	
Subjective social status, mean (SD)	4.28 (1.88)	
Self-rated health, mean (SD)	3.36 (1.13)	
Female, *n* (%)	3,015 (54.0%)	
Urban residence, *n* (%)	3,043 (54.5%)	
Agricultural hukou, *n* (%)	3,824 (68.5%)	
Married/cohabiting, *n* (%)	4,121 (73.8%)	
Telephone interview, *n* (%)	324 (5.8%)	

Unweighted estimates describe the analytical sample (*N* = 5,585). Weighted percentages in Panel A incorporate the CGSS2023 integrated survey weight and the item-observation adjustment. Values are mean (SD), median [IQR], or *n* (%).

The 324 telephone respondents contributed 47.6% of the total combined weight. The weight-based effective sample size was 968, compared with 4,368 using the integrated survey weight alone. In the face-to-face sample with household weights, dual engagement accounted for 11.0%, compared with 21.9% in the full sample with combined weights (Supplementary Table S5). Compared with included respondents, those excluded at the complete-case stage more often reported live attendance (27.6% vs. 22.7%) and were more often interviewed by telephone (10.2% vs. 5.8%; Supplementary Table S3, Panel A).

### 4.2 Unadjusted socioeconomic distribution of joint sport-engagement categories

With combined weights, low engagement declined from 82.7% among respondents with no schooling to 10.3% among those with postgraduate education, while dual engagement increased from 1.3% to 40.2% (Figure 2). Across income quartiles, spectator-only engagement rose from 7.9% in Q1 to 23.3% in Q4 and dual engagement from 17.6% to 30.8%, although neither category followed a monotonic pattern. Differences by subjective social status were more modest. Low engagement accounted for 59.1% of rural respondents and 31.9% of urban respondents, whereas the corresponding proportions for dual engagement were 11.3% and 27.6%.

**Figure 2:**
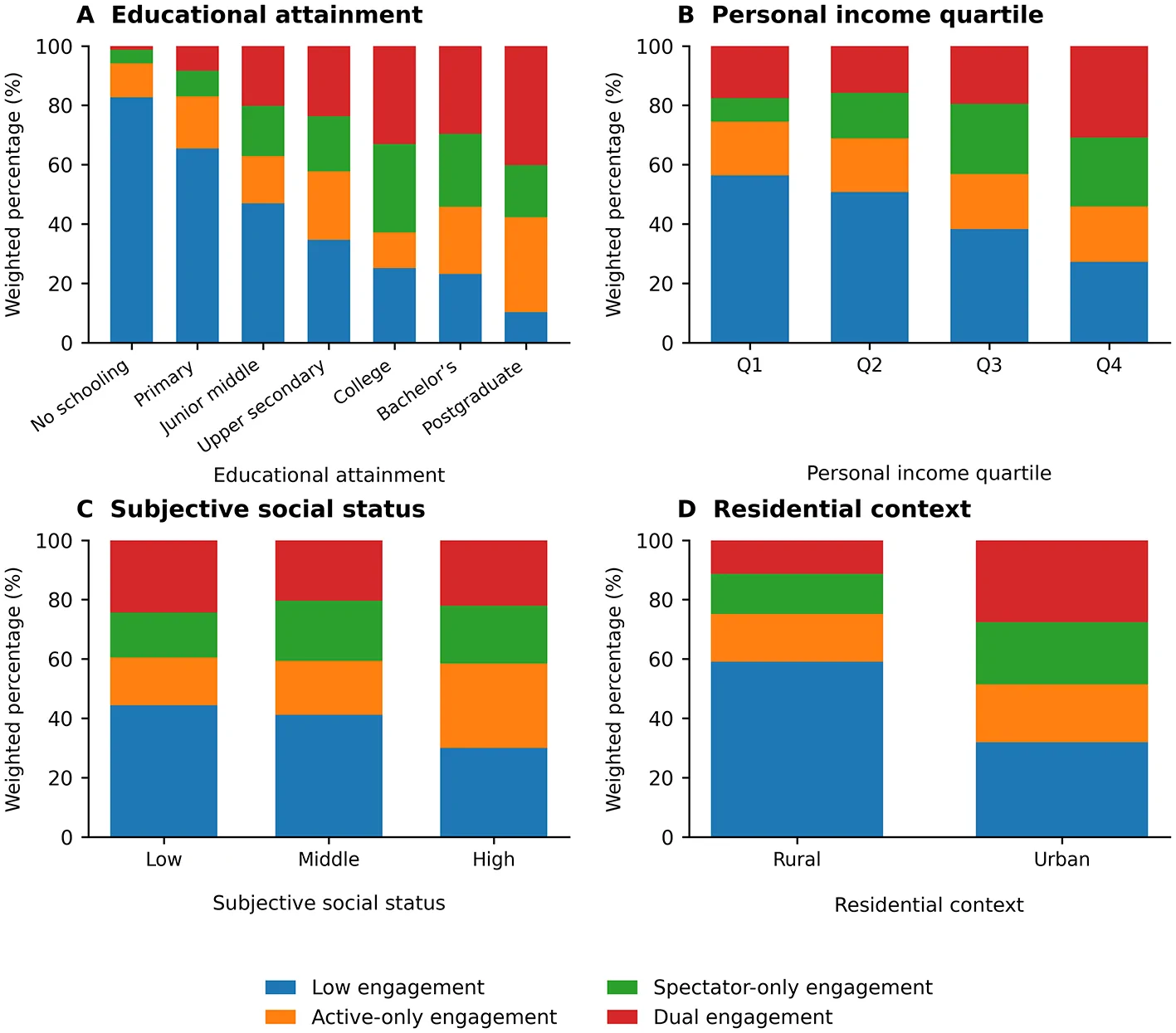
Unadjusted distribution of joint sport-engagement categories across socioeconomic groups, using combined weights. **(A)** Educational attainment; **(B)** personal income quartile; **(C)** subjective social status; **(D)** residential context. Percentages sum to 100 within groups; Q1–Q4 denote income quartiles.

### 4.3 Adjusted associations of socioeconomic indicators with joint sport-engagement categories

In the primary unweighted model, education was positively associated with all three non-reference categories, with the largest relative risk ratio for dual engagement (RRR = 1.185, 95% CI 1.148–1.224; Table 2). Higher log-transformed income was associated with a lower relative likelihood of active-only engagement and a higher relative likelihood of spectator-only engagement, but showed no clear association with dual engagement. Subjective social status was positively associated with all three non-reference categories. Urban residence was also positively associated with all three, with the largest urban–rural contrast for dual engagement (RRR = 2.287, 95% CI 1.753–2.985). The primary estimates supported H1 and H3. Support for H2 varied with income specification and covariate adjustment. Support for H4 was limited to the primary unweighted and face-to-face-only models.

**Table 2:** Adjusted relative risk ratios for joint sport-engagement categories: unweighted and combined-weight models.

Predictor contrast	Active-only engagement RRR (95% CI)	Spectator-only engagement RRR (95% CI)	Dual engagement RRR (95% CI)
Panel A. Primary unweighted model (*N* = 5,585)			
Education, per 1-unit years-equivalent increase	1.104 (1.081–1.128)	1.072 (1.040–1.105)	1.185 (1.148–1.224)
Personal income, per doubling of (income + 1)	0.984 (0.971–0.997)	1.027 (1.006–1.048)	0.995 (0.977–1.014)
Subjective social status, per 1-rung increase	1.066 (1.027–1.107)	1.095 (1.042–1.152)	1.100 (1.044–1.159)
Urban vs. rural residence	1.465 (1.223–1.755)	1.500 (1.182–1.904)	2.287 (1.753–2.985)
Panel B. Combined survey/item-weight model (*N* = 5,585)			
Education, per 1-unit years-equivalent increase	1.147 (1.095–1.201)	1.062 (0.995–1.133)	1.190 (1.112–1.273)
Personal income, per doubling of (income + 1)	0.991 (0.963–1.020)	1.047 (1.005–1.091)	0.989 (0.947–1.032)
Subjective social status, per 1-rung increase	1.119 (1.023–1.225)	1.194 (1.063–1.341)	1.037 (0.924–1.164)
Urban vs. rural residence	1.092 (0.750–1.591)	0.851 (0.505–1.434)	1.412 (0.801–2.488)

Values are relative risk ratios (RRRs) with 95% confidence intervals (CIs). Both models use low engagement as the reference and include all four focal predictors and the covariates in Section 3.5. Panel B uses combined weights (Supplementary Table S5). Income RRRs are per doubling of (income + 1).

On the probability scale, the model-based average marginal effects of the years-equivalent education score were −2.20 percentage points (pp) for low engagement, +1.02 pp for active-only engagement and +1.09 pp for dual engagement. The spectator-only estimate was close to zero (+0.09 pp, 95% CI −0.17 to 0.35). Comparing annual incomes of 3,000 and 50,000 yuan, adjusted probabilities differed by −1.32 pp for active-only and +1.21 pp for spectator-only engagement (Supplementary Table S7, Panel E). Higher subjective social status was associated with a lower probability of low engagement and small increases in the other categories. Compared with rural residence, urban residence corresponded to a 10.19-pp lower probability of low engagement and 3.37- and 5.33-pp higher probabilities of active-only and dual engagement, respectively; the spectator-only confidence interval included zero (Figure 3).

**Figure 3:**
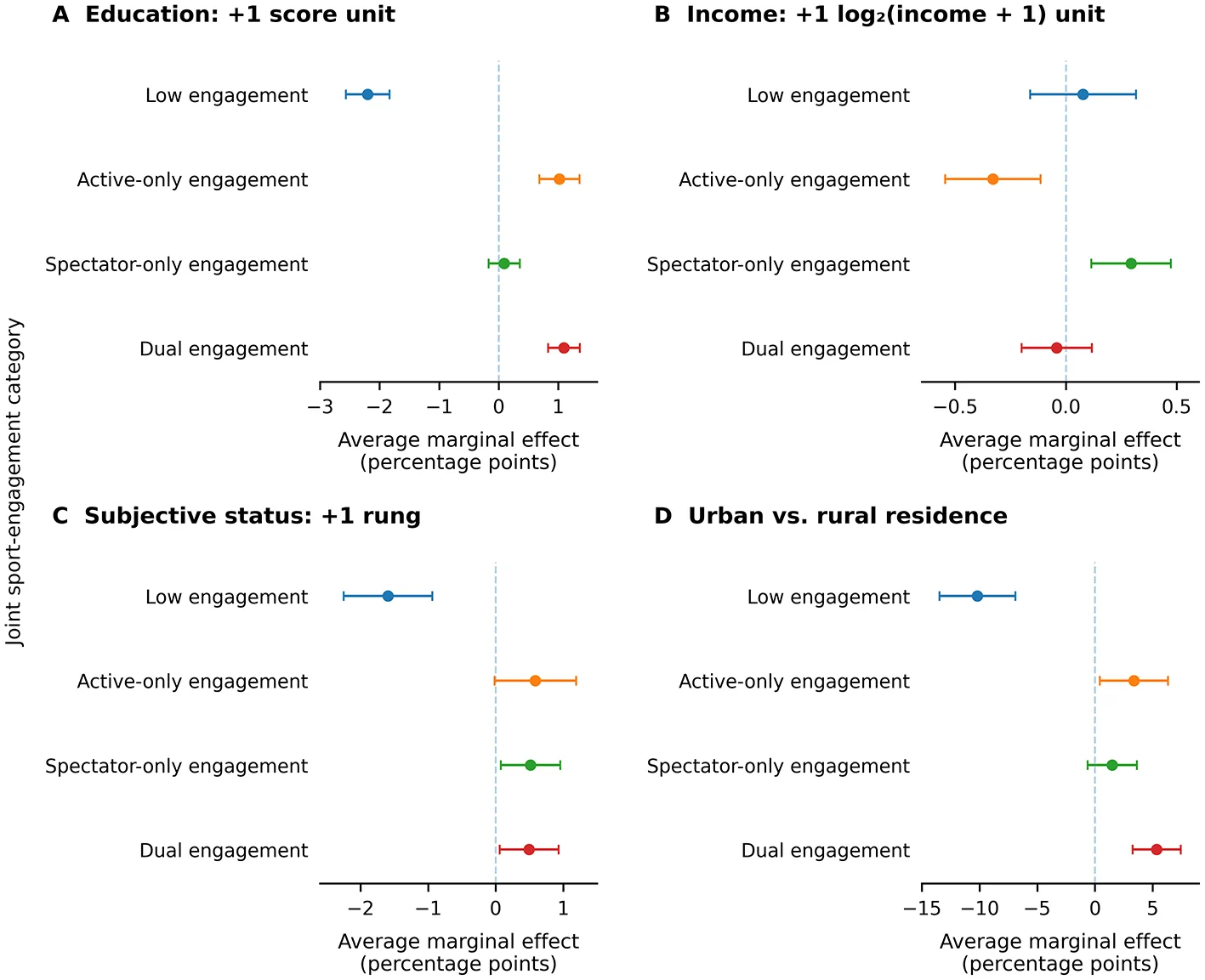
Adjusted average marginal effects on joint sport-engagement category probabilities. **(A)** Education; **(B)** personal income; **(C)** subjective social status; **(D)** urban vs. rural residence. Points show AMEs from the primary unweighted model and bars show 95% CIs. X-axis scales vary across panels.

The urban–rural RRR for dual engagement was 1.412 (95% CI 0.801–2.488) in the combined-weight model, compared with 2.287 in the primary unweighted model (Table 2). The selection-adjusted estimate was 1.458, and none of the three urban–rural contrasts was statistically significant in either weighted model (Supplementary Table S8). Education remained positively associated with active-only and dual engagement in the combined-weight model, whereas the inverse income association with active-only engagement and the positive subjective-status association with dual engagement were no longer statistically significant.

In the primary unweighted model, the telephone-vs.-face-to-face RRRs were 4.082 (95% CI 2.144–7.771) for spectator-only engagement and 6.826 (95% CI 3.624–12.857) for dual engagement, each compared with low engagement (Supplementary Table S4). In the unweighted face-to-face-only model, the RRRs for dual engagement were 1.187 (95% CI 1.146–1.229) for education and 2.429 (95% CI 1.835–3.216) for urban vs. rural residence (Supplementary Table S8, Panel B). In the household-weighted face-to-face model, the urban–rural RRRs were 1.525 for spectator-only and 1.984 for dual engagement (both *p* < 0.05; Supplementary Table S8, Panel A).

### 4.4 Sensitivity to variable coding, work status and outcome definitions

Joint Wald tests did not reject the linear education-score restrictions (unweighted: χ^2^ = 13.87, df = 15, *p* = 0.536; combined-weight: χ^2^ = 23.95, df = 15, *p* = 0.066; Supplementary Table S7). In the unweighted categorical model, adjusted dual-engagement probabilities were 18.1% for college, 16.8% for bachelor's and 16.2% for postgraduate education. The postgraduate–bachelor's contrasts were imprecise in both models; the postgraduate group contained 78 respondents (Supplementary Table S7, Panels A1–A4).

When zero income was modeled separately, the positive-income term for active-only engagement was not statistically significant under either the unweighted or combined-weight specification. Among respondents with positive incomes, income was positively associated with spectator-only engagement in the unweighted model and with dual engagement in both models (Supplementary Table S7, Panel H).

After work-status adjustment in 5,256 face-to-face respondents, the ln (income + 1) association with active-only engagement remained statistically significant only with household weights; its association with spectator-only engagement was not statistically significant under either weighting specification. When zero income was also modeled separately, the positive-income association with spectator-only engagement remained statistically significant only in the unweighted model (RRR = 1.110, 95% CI 1.024–1.203). The positive-income terms for active-only and dual engagement were not statistically significant under either weighting specification (Supplementary Table S8, Panel C).

In the original five-category analyses, education was positively associated with each exercise frequency vs. never in both unweighted and combined-weight models (Table 3). Income was positively associated with attendance several times a year or less; confidence intervals for higher frequencies included one. Only 29 respondents reported daily attendance.

**Table 3:** Adjusted associations with the original five-category exercise and live sport attendance frequencies.

Past-year frequency	*n*	Model	Adjusted RRR (95% CI)			
			Education	Personal income	Subjective social status	Urban vs. rural
Panel A. Leisure-time exercise						
Never (reference)	2,296	Both	Reference	Reference	Reference	Reference
Several times a year or less	690	Unweighted	1.089 (1.060–1.120)	1.006 (0.988–1.025)	1.124 (1.070–1.181)	2.347 (1.883–2.925)
		Combined- weight	1.097 (1.030–1.168)	1.018 (0.979–1.059)	1.186 (1.060–1.326)	1.781 (1.053–3.011)
Several times a month	643	Unweighted	1.148 (1.112–1.186)	1.024 (1.003–1.044)	1.103 (1.046–1.163)	2.480 (1.944–3.164)
		Combined- weight	1.232 (1.151–1.318)	1.034 (0.988–1.083)	1.123 (0.991–1.273)	2.550 (1.587–4.098)
Several times a week	792	Unweighted	1.182 (1.149–1.216)	0.989 (0.973–1.006)	1.127 (1.073–1.184)	2.241 (1.793–2.800)
		Combined- weight	1.248 (1.169–1.333)	0.992 (0.953–1.032)	1.124 (1.010–1.251)	2.150 (1.292–3.579)
Daily	1,164	Unweighted	1.156 (1.129–1.184)	0.983 (0.968–0.997)	1.092 (1.047–1.138)	2.110 (1.748–2.547)
		Combined- weight	1.233 (1.172–1.297)	0.972 (0.938–1.008)	1.108 (1.018–1.206)	1.438 (0.947–2.183)
Panel B. Live sport attendance						
Never (reference)	4,318	Both	Reference	Reference	Reference	Reference
Several times a year or less	932	Unweighted	1.092 (1.065–1.120)	1.020 (1.004–1.036)	1.061 (1.016–1.107)	1.663 (1.368–2.021)
		Combined- weight	1.079 (1.022–1.138)	1.036 (1.001–1.072)	1.037 (0.936–1.148)	1.048 (0.692–1.587)
Several times a month	203	Unweighted	1.047 (1.001–1.096)	1.014 (0.988–1.041)	1.035 (0.954–1.124)	1.490 (1.031–2.153)
		Combined- weight	1.078 (0.978–1.188)	0.983 (0.932–1.035)	0.959 (0.804–1.144)	1.489 (0.663–3.344)
Several times a week	103	Unweighted	1.065 (1.011–1.122)	0.981 (0.946–1.016)	1.066 (0.955–1.189)	1.285 (0.755–2.187)
		Combined- weight	0.973 (0.889–1.064)	0.946 (0.869–1.029)	1.203 (1.007–1.436)	0.956 (0.336–2.716)
Daily	29	Unweighted	1.175 (1.042–1.326)	1.021 (0.931–1.120)	1.232 (1.040–1.460)	0.789 (0.295–2.108)
		Combined- weight	1.225 (1.061–1.415)	1.064 (0.946–1.197)	1.576 (1.145–2.169)	0.305 (0.054–1.730)

*N* = 5,585; n denotes unweighted counts. Separate multinomial models include all focal predictors and prespecified covariates except province. Education and subjective-status RRRs are per years-equivalent unit and ladder rung, respectively; income RRRs are per doubling of (income + 1). Confidence intervals use respondent-level robust standard errors. RRR, relative risk ratio; CI, confidence interval.

In the unweighted sample, 79.4% of spectator-only and 67.6% of dual-engagement respondents attended several times a year or less. With attendance defined as at least several times a month, education remained positively associated with active-only and dual engagement in both models. Income RRRs for spectator-only engagement were near one, whereas the combined-weight model showed an inverse income association with dual engagement (RRR = 0.927, 95% CI 0.877–0.980; Supplementary Table S6, Panels A and E).

Within low engagement, 829 of 2,992 respondents (27.7%) exercised occasionally. Separating them from never-exercisers changed the reported adjusted probability contrasts for the other three categories by at most 0.32 percentage points unweighted and 0.57 with combined weights, compared with the four-category model using the same adjustment set (Supplementary Table S6, Panels D and G).

### 4.5 Exploratory heterogeneity

Of the nine exploratory interactions, only education × residence remained significant after false-discovery-rate adjustment (omnibus Wald χ^2^ = 29.16, df = 3, raw *p* < 0.001, BH-adjusted *q* < 0.001; Table 4). The other eight interactions had q ≥ 0.05.

**Table 4:** Omnibus tests of exploratory interactions.

Socioeconomic indicator	Modifier	Wald chi-square	df	Raw *p*	BH-adjusted *q*
Education	Sex	7.36	3	0.061	0.138
	Age group	1.81	3	0.614	0.690
	**Residence**	**29.16**	**3**	**< 0.001**	**< 0.001**
Income	Sex	3.65	3	0.302	0.389
	Age group	8.30	3	0.040	0.137
	Residence	5.12	3	0.163	0.289
Subjective status	Sex	4.73	3	0.193	0.289
	Age group	0.21	3	0.975	0.975
	Residence	8.02	3	0.046	0.137

*q* values are Benjamini–Hochberg adjusted across the nine tests; the sole *q* < 0.05 result is bold.

In the unweighted linear-score model, the education RRR for dual vs. low engagement was 1.317 (95% CI 1.243–1.395) per score unit in rural respondents and 1.131 (95% CI 1.092–1.171) in urban respondents (Supplementary Table S9, Panel F1). The six-level categorical interaction was statistically significant in the unweighted model (χ^2^ = 44.69, df = 15, *p* < 0.001), but not with combined weights (χ^2^ = 8.66, df = 15, *p* = 0.895). Tests of the matched linear-score restrictions gave *p* = 0.366 and *p* = 0.243, respectively (Supplementary Table S9, Panel G2).

For dual engagement, unweighted urban–rural probability differences varied non-monotonically across education levels: 4.77 pp at primary education, 9.01 pp at junior middle and −3.21 pp at bachelor's or higher. All six combined-weight confidence intervals included zero (Figure 4; Supplementary Table S9, Panels H1–H2).

**Figure 4:**
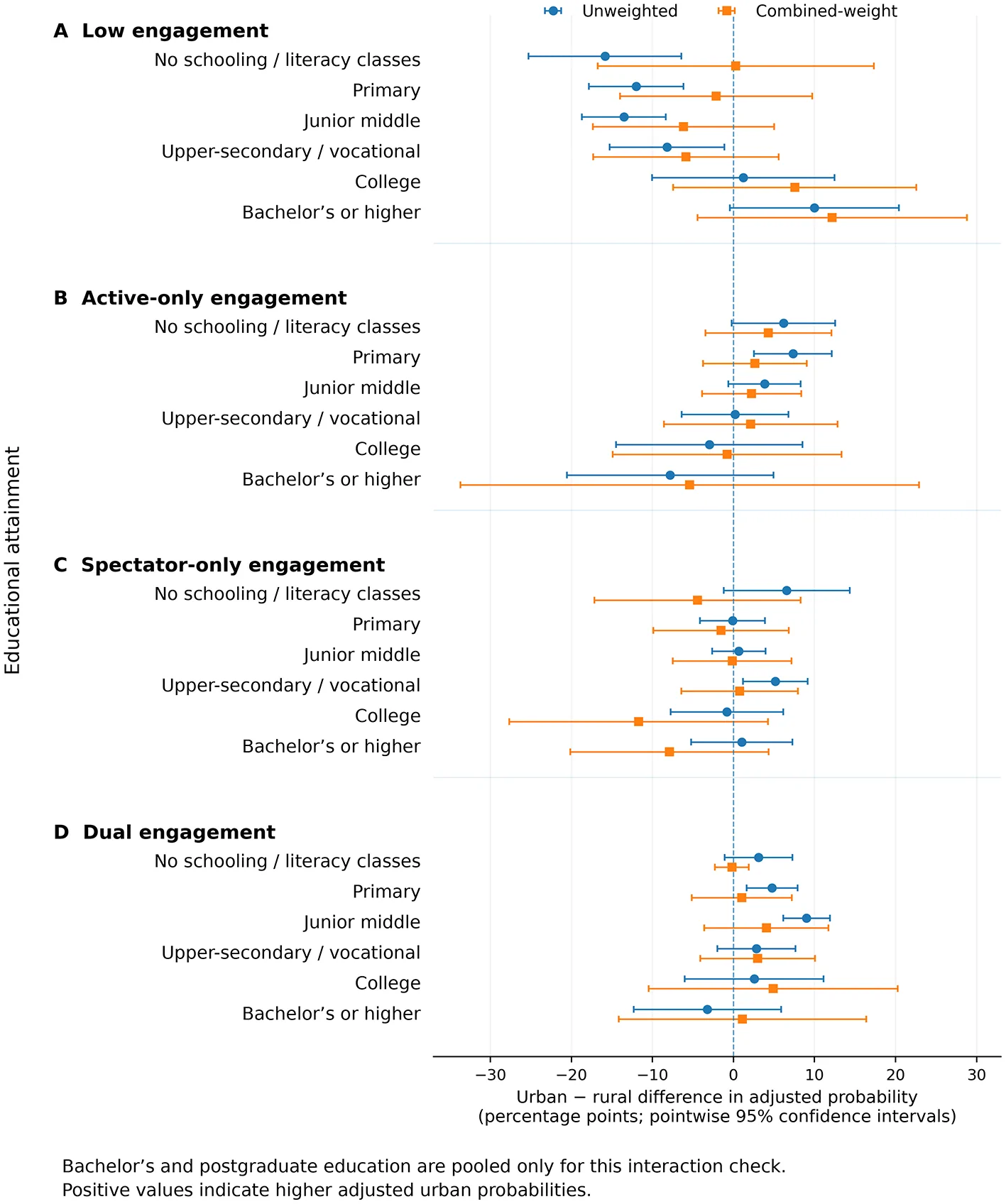
Adjusted urban–rural differences in joint sport-engagement probabilities by education. **(A)** Low engagement; **(B)** active-only engagement; **(C)** spectator-only engagement; **(D)** dual engagement. Points and bars show urban-minus-rural differences (percentage points) and pointwise 95% CIs from unweighted and combined-weight categorical education-by-residence models. Bachelor's and postgraduate education are pooled for this interaction check (Supplementary Table S9, Panels H1–H2).

## 5 Discussion

The joint categories showed that socioeconomic differences did not fall evenly across exercise and live attendance. Education was the most consistent correlate, particularly for active-only and dual engagement. Income associations varied across model specifications and attendance definitions. Subjective social status showed modest differences, while urban–rural findings depended on the weighting specification.

The contrast between education and income is informative because the two capture different resources. Education may shape sporting socialization, familiarity with organized activity and routines carried across the life course, which can make frequent exercise easier to sustain (15, 31–33). Income is more directly tied to discretionary spending and the immediate costs of attendance (9, 21, 22, 34, 35). The inverse association with active-only engagement depended on zero-income coding, and work-status adjustment affected the income findings differently across weighting specifications. Associations with subjective social status were modest after adjustment for education, income and the other covariates. The different association patterns support considering these socioeconomic indicators separately.

Residence introduced a different dimension. Urban residence was positively associated with dual engagement in the primary unweighted model, whereas the combined-weight and selection-adjusted estimates were smaller and not statistically significant. Differences in access to facilities, transport, organized activities and sporting events offer a possible explanation (18, 23, 24, 36, 37). These local conditions were not assessed directly and may remain sources of confounding after province adjustment. The categorical education-by-residence interaction was statistically significant unweighted but not with combined weights.

Monitoring and program evaluation should distinguish exercise from live attendance while also recording whether they occur together. This preserves the distinction between active and spectator involvement in sport.

The cross-sectional design limits causal interpretation, and the items measure frequency rather than duration, intensity or activity type. Personal income does not capture shared household resources, and work status refers to the interview period rather than the income reference year. Respondents doing agricultural or other manual work may report little leisure-time exercise despite substantial activity at work. Work-status categories do not quantify occupational activity, leaving scope for residual confounding. Differences in how respondents interpret “exercise” may also affect urban–rural comparisons. The attendance item cannot distinguish one-off visits from other low-frequency participation. The combined weights gave substantial influence to the small telephone subsample. Interview-mode differences may reflect sample composition, reporting or both. Mode-related reporting error and selection related to incompletely observed or unmeasured characteristics could affect both weighted percentages and regression estimates.

## 6 Conclusion

In the CGSS2023 analytical sample, socioeconomic associations differed across combinations of frequent leisure-time exercise and live sport attendance. Education showed the most consistent associations, particularly with active-only and dual engagement. Income associations depended on model specification, subjective-status differences were modest, and evidence for urban–rural differences depended on weighting. The joint classification located these associations within specific combinations of exercise and attendance. Population monitoring and program evaluation should therefore report the two behaviors separately while also examining whether they occur together.

## Data Availability

Publicly available datasets were analyzed in this study. This data can be found here: Chinese General Social Survey 2023 (CGSS2023), Chinese National Survey Data Archive (CNSDA), Project ID 11256439: https://www.cnsda.org/index.php?id=11256439&r=projects/view.
