# Supplementary material for: Socioeconomic differentiation in sport engagement: frequent exercise, live sport attendance, and their overlap in the 2023 Chinese General Social Survey
Source: Front Public Health. 2026 Oct 7;14:1965653. doi: 10.3389/fpubh.2026.1965653 (PMC13682996; doi:10.3389/fpubh.2026.1965653)
Supplement: Supplementary file 1 [file Table_1.docx]

**Supplementary material**

# **Supplementary Table S1. Variables, source items, and analytical coding**

| **Variable** | **CGSS item** | **Analytical coding** |
| --- | --- | --- |
| Frequent exercise | a3009 | Past-year leisure-time exercise: 1 = every day or several times a week; 0 = several times a month, several times a year or less, or never. |
| Live attendance | a3010 | 1 = any live sporting-event attendance in the previous year; 0 = never. |
| Joint sport-engagement category | a3009 + a3010 | Low engagement: neither frequent exercise nor live attendance; active-only: frequent exercise without attendance; spectator-only: attendance without frequent exercise; dual engagement: both. Nominal outcome; low engagement is the model reference. |
| Education | a7a | Years-equivalent: 0 (no schooling or literacy classes), 6 (primary), 9 (junior middle), 12 (upper-secondary or vocational), 15 (college), 16 (bachelor’s), and 19 (postgraduate); other and non-substantive responses are missing. |
| Log-transformed personal income | a8a | Total personal income in 2022 (yuan). Observed nonnegative amounts are retained, including values above 1,000,000 yuan. The special top-code (9,999,996), negative values and non-substantive responses are missing. Primary-model term: ln(income + 1); the constant is one yuan. |
| Zero-income indicator | a8a | Sensitivity models: 1 = zero personal income; 0 = positive personal income. Entered jointly with the positive-income log term; its RRR compares zero income with 20,000 yuan (Table S7, Panel H; Table S8, Panel C). |
| Positive-income log term | a8a | Sensitivity models: ln(income/20,000) for positive income and 0 for zero income. Entered jointly with the zero-income indicator. RRRs for this term are per doubling of positive income; 20,000 yuan is the unweighted analytical-sample median. |
| Work status | a53, a54 | Face-to-face sensitivity models using previous-week status: working or temporarily absent (reference), a53 = 2, 3 or 4; retired and not working, a53 = 1 and a54 = 7; otherwise not working, a53 = 1 and a54 = 1–6, 8 or 9. Other responses are missing. Telephone interviews did not administer a53–a54. |
| Subjective social status | a43a | Continuous 1–10 score; higher values indicate a higher self-placement. |
| Urban residence | a25a | 1 = city center, urban fringe/transition zone, or town; 0 = rural village. |
| Age | a3a | 2023 minus birth year; restricted to ages 18–100; centered at 50, with a quadratic term. |
| Female | a2 | 1 = female; 0 = male. |
| Self-rated health | a15 | 1 = very unhealthy to 5 = very healthy. |
| Agricultural hukou | a18 | 1 = agricultural hukou or resident hukou formerly agricultural; 0 = non-agricultural hukou or resident hukou formerly non-agricultural; other categories missing. |
| Partnered | a69 | 1 = cohabiting, first marriage with spouse, or remarriage with spouse; 0 = other valid status. |
| Survey mode | dsource | 1 = telephone; 0 = face-to-face. |
| Province | s41 | Province indicators; codes with fewer than 20 complete cases pooled into one sparse-province category. |

Note. Non-substantive, inapplicable and out-of-range responses are missing. Education scores denote attainment, not completed years. Only the categorical education × residence check pools bachelor’s and postgraduate levels (Table S9, G1); the primary analysis and Table S7 retain seven levels.

# **Supplementary Table S2. Variable-specific missingness and sample-selection counts**

## **Panel A. Item-level and joint validity among age-eligible respondents**

| **Variable** | **All (N = 11,242) Valid / missing (%)** | **Face-to-face (N = 5,583) Valid / missing (%)** | **Telephone (N = 5,659) Valid / missing (%)** |
| --- | --- | --- | --- |
| Frequent exercise item | 6,983 / 4,259 (37.9%) | 5,573 / 10 (0.2%) | 1,410 / 4,249 (75.1%) |
| Live attendance item | 6,941 / 4,301 (38.3%) | 5,554 / 29 (0.5%) | 1,387 / 4,272 (75.5%) |
| Both sport items (joint validity) | 5,908 / 5,334 (47.4%) | 5,551 / 32 (0.6%) | 357 / 5,302 (93.7%) |
| Education | 11,237 / 5 (0.0%) | 5,582 / 1 (0.0%) | 5,655 / 4 (0.1%) |
| Personal income | 10,569 / 673 (6.0%) | 5,359 / 224 (4.0%) | 5,210 / 449 (7.9%) |
| Subjective social status | 11,132 / 110 (1.0%) | 5,530 / 53 (0.9%) | 5,602 / 57 (1.0%) |
| Residential context | 11,175 / 67 (0.6%) | 5,562 / 21 (0.4%) | 5,613 / 46 (0.8%) |
| Sex | 11,242 / 0 (0.0%) | 5,583 / 0 (0.0%) | 5,659 / 0 (0.0%) |
| Self-rated health | 11,238 / 4 (0.0%) | 5,581 / 2 (0.0%) | 5,657 / 2 (0.0%) |
| Household registration | 11,178 / 64 (0.6%) | 5,575 / 8 (0.1%) | 5,603 / 56 (1.0%) |
| Marital/cohabitation status | 11,239 / 3 (0.0%) | 5,583 / 0 (0.0%) | 5,656 / 3 (0.1%) |
| Survey mode | 11,242 / 0 (0.0%) | 5,583 / 0 (0.0%) | 5,659 / 0 (0.0%) |
| Province | 11,237 / 5 (0.0%) | 5,583 / 0 (0.0%) | 5,654 / 5 (0.1%) |

Note. Denominators are age-eligible respondents; parentheses give missing percentages. Missingness includes randomized telephone-item non-administration, non-substantive responses and invalid codes.

## **Panel B. Sequential sample selection**

| **Selection step** | **Remaining N** | **Face-to-face N** | **Telephone N** | **Removed at step** |
| --- | --- | --- | --- | --- |
| Released CGSS2023 sample | 11,326 | 5,583 | 5,743 | — |
| Age 18–100 years | 11,242 | 5,583 | 5,659 | 84 |
| Valid exercise and attendance items | 5,908 | 5,551 | 357 | 5,334 |
| Complete focal socioeconomic indicators | 5,595 | 5,269 | 326 | 313 |
| Complete prespecified covariates | 5,585 | 5,261 | 324 | 10 |

Note. Among 5,908 respondents with valid outcomes, missing focal indicators were income (250), subjective status (53), residence (24) and education (1); counts overlap. The primary analysis used complete cases without imputation.

# **Supplementary Table S3. Comparisons by analytical inclusion and telephone item availability**

## **Panel A. Included and excluded respondents with valid joint-outcome data**

| **Characteristic** | **Included (N = 5,585)** | | **Excluded (N = 323)** | |
| --- | --- | --- | --- | --- |
|  | **Observed n** | **Summary** | **Observed n** | **Summary** |
| Age, years | 5,585 | 53.94 (16.44) | 323 | 51.46 (17.45) |
| Female | 5,585 | 54.0% | 323 | 49.2% |
| Education, years-equivalent | 5,585 | 9.31 (4.64) | 322 | 9.19 (5.17) |
| Personal income, yuan | 5,585 | 20,000 [3,000–50,000] | 73 | 20,000 [1,200–50,000] |
| Subjective social status | 5,585 | 4.28 (1.88) | 270 | 4.06 (1.98) |
| Urban residence | 5,585 | 54.5% | 299 | 50.5% |
| Self-rated health | 5,585 | 3.36 (1.13) | 321 | 3.32 (1.16) |
| Agricultural or formerly agricultural hukou | 5,585 | 68.5% | 313 | 70.9% |
| Partnered | 5,585 | 73.8% | 323 | 70.6% |
| Telephone interview | 5,585 | 5.8% | 323 | 10.2% |
| Frequent exercise | 5,585 | 35.0% | 323 | 35.3% |
| Any live attendance | 5,585 | 22.7% | 323 | 27.6% |
| Category: low engagement | 5,585 | 53.6% | 323 | 50.2% |
| Category: active-only | 5,585 | 23.7% | 323 | 22.3% |
| Category: spectator-only | 5,585 | 11.4% | 323 | 14.6% |
| Category: dual engagement | 5,585 | 11.3% | 323 | 13.0% |

Note. Unweighted means (SD), medians [interquartile range] or percentages among 5,908 respondents with valid outcomes, using variable-specific observed counts.

## **Panel B. Item availability among age-eligible telephone respondents**

| **Characteristic** | **Both items valid (N = 357)** | | **At least one item missing (N = 5,302)** | |
| --- | --- | --- | --- | --- |
|  | **Observed n** | **Summary** | **Observed n** | **Summary** |
| Age, years | 357 | 42.11 (14.38) | 5,302 | 40.92 (14.12) |
| Female | 357 | 30.5% | 5,302 | 35.5% |
| Education, years-equivalent | 357 | 13.81 (3.45) | 5,298 | 13.90 (3.33) |
| Personal income, yuan | 329 | 60,000 [30,000–120,000] | 4,881 | 60,000 [30,000–120,000] |
| Subjective social status | 356 | 4.15 (2.00) | 5,246 | 4.29 (1.85) |
| Urban residence | 354 | 87.0% | 5,259 | 85.0% |
| Self-rated health | 357 | 3.44 (0.97) | 5,300 | 3.42 (0.93) |
| Agricultural or formerly agricultural hukou | 355 | 47.3% | 5,248 | 54.2% |
| Partnered | 357 | 71.4% | 5,299 | 65.7% |

Note. All 5,659 age-eligible telephone respondents are included before complete-case exclusions. Summaries follow Panel A; no imputation.

# **Supplementary Table S4. Primary multinomial logistic regression results**

| **Predictor** | **Outcome versus low engagement** | **RRR** | **95% CI** | **p** |
| --- | --- | --- | --- | --- |
| Education | Active-only engagement | 1.104 | 1.081–1.128 | <0.001 |
|  | Spectator-only engagement | 1.072 | 1.040–1.105 | <0.001 |
|  | Dual engagement | 1.185 | 1.148–1.224 | <0.001 |
| Personal income, per 1-unit increase in ln(income + 1) | Active-only engagement | 0.977 | 0.958–0.996 | 0.016 |
|  | Spectator-only engagement | 1.039 | 1.009–1.069 | 0.010 |
|  | Dual engagement | 0.993 | 0.967–1.021 | 0.633 |
| Subjective social status | Active-only engagement | 1.066 | 1.027–1.107 | <0.001 |
|  | Spectator-only engagement | 1.095 | 1.042–1.152 | <0.001 |
|  | Dual engagement | 1.100 | 1.044–1.159 | <0.001 |
| Urban residence | Active-only engagement | 1.465 | 1.223–1.755 | <0.001 |
|  | Spectator-only engagement | 1.500 | 1.182–1.904 | <0.001 |
|  | Dual engagement | 2.287 | 1.753–2.985 | <0.001 |
| Age centered at 50, per 10 years | Active-only engagement | 1.260 | 1.188–1.336 | <0.001 |
|  | Spectator-only engagement | 0.751 | 0.694–0.813 | <0.001 |
|  | Dual engagement | 1.061 | 0.985–1.143 | 0.120 |
| Centered age squared / 100 | Active-only engagement | 0.953 | 0.928–0.979 | <0.001 |
|  | Spectator-only engagement | 0.967 | 0.928–1.007 | 0.106 |
|  | Dual engagement | 0.988 | 0.953–1.023 | 0.492 |
| Female | Active-only engagement | 0.839 | 0.727–0.968 | 0.016 |
|  | Spectator-only engagement | 0.664 | 0.551–0.801 | <0.001 |
|  | Dual engagement | 0.552 | 0.452–0.673 | <0.001 |
| Self-rated health | Active-only engagement | 1.219 | 1.139–1.304 | <0.001 |
|  | Spectator-only engagement | 1.105 | 1.008–1.213 | 0.034 |
|  | Dual engagement | 1.416 | 1.284–1.563 | <0.001 |
| Agricultural hukou | Active-only engagement | 0.800 | 0.665–0.964 | 0.019 |
|  | Spectator-only engagement | 0.784 | 0.621–0.989 | 0.040 |
|  | Dual engagement | 0.866 | 0.682–1.099 | 0.235 |
| Partnered | Active-only engagement | 0.895 | 0.756–1.059 | 0.197 |
|  | Spectator-only engagement | 1.176 | 0.917–1.509 | 0.201 |
|  | Dual engagement | 0.892 | 0.701–1.135 | 0.352 |
| Telephone | Active-only engagement | 1.833 | 0.960–3.500 | 0.066 |
|  | Spectator-only engagement | 4.082 | 2.144–7.771 | <0.001 |
|  | Dual engagement | 6.826 | 3.624–12.857 | <0.001 |

Note. N = 5,585; unweighted; reference: low engagement. Predictors and pooled province indicators enter jointly; province coefficients are omitted. Education/status RRRs are per score unit/rung. Income RRRs are per unit of ln(income + 1); main-text Table 2 uses ln(2) units. HC0 denotes respondent-level robust sandwich covariance without small-sample correction. RRR, relative risk ratio; CI, confidence interval.

# **Supplementary Table S5. Survey weighting and sample-selection diagnostics**

## **Panel A. Weight construction**

| **Component** | **Definition** | **Use** | **Implementation** |
| --- | --- | --- | --- |
| Integrated survey weight | weight2 | Combined-weight descriptive and regression analyses | Released integrated CGSS2023 weight |
| Household-interview weight | weight1 | Face-to-face weighted analyses | Normalized to mean 1 in the face-to-face analytical sample |
| A30 observation adjustment | 1 / p_m_ | Combined with weight2 | Face-to-face: p_m_ = 5,551/5,583; telephone: p_m_ = 357/5,659. |
| Complete-case selection adjustment | 1 / estimated probability of complete-case inclusion | Selection-IPW sensitivity model | Divide the combined weight by the fitted inclusion probability; normalize, cap at the 1st/99th percentiles and renormalize to mean 1. |

Note. p_m is joint-item validity within each age-eligible interview-mode sample before complete-case exclusions. Adjustment assumes that valid cases represent the within-mode outcome distribution after survey weighting.

**Complete-case inclusion model.** We re-estimated Pr(R = 1) by unweighted binary logistic regression in all 5,908 age-eligible respondents with both sport items valid: R = 1 for 5,585 primary-model complete cases and R = 0 for 323 excluded cases. Predictors were (age − 50)/10, (age − 50)²/100, sex, partnered status, survey mode, three joint-category indicators (reference: low engagement) and 19 pooled-province indicators (reference: the original sparse-province group; Table S9, Panel E). Binary coding follows Table S1. All predictors were observed in all 5,908 cases; no imputation or further exclusions were used. Incompletely observed education, income, subjective status, residence, health and hukou were not used in the inclusion model; they remained in the outcome model.

**Weight application and diagnostics.** For complete cases, we divided the combined weight by the fitted inclusion probability, normalized to mean one, capped weights at the empirical 1st and 99th percentiles (0.199008 and 13.210373; linear interpolation), then renormalized to mean one. Capping affected 56 weights at each tail; no cases were removed. The inclusion model converged with fitted probabilities of 0.712–0.990. This analysis cannot exclude selection related to incompletely observed socioeconomic measures or unobserved characteristics.

## **Panel B. Weight distributions**

| **Weight** | **N** | **Mean** | **SD** | **Min** | **Median** | **P99** | **Max** | **Effective N** |
| --- | --- | --- | --- | --- | --- | --- | --- | --- |
| Integrated survey weight (weight2), analytical sample | 5,585 | 1.022 | 0.539 | 0.071 | 0.912 | 2.161 | 2.161 | 4,368 |
| Combined survey/item-observation weight | 5,585 | 1.000 | 2.185 | 0.175 | 0.509 | 13.068 | 18.464 | 968 |
| Household-interview weight (weight1), face-to-face sample | 5,261 | 1.000 | 0.686 | 0.290 | 0.796 | 2.817 | 2.817 | 3,577 |
| Selection-IPW combined weight, before trimming | 5,585 | 1.000 | 2.256 | 0.173 | 0.495 | 13.210 | 21.948 | 917 |
| Selection-IPW combined weight, after 1st/99th percentile trimming | 5,585 | 1.000 | 2.123 | 0.205 | 0.510 | 13.604 | 13.610 | 1,014 |

Note. N is the weight-summary sample. Effective N = (sum of weights)² / sum of squared weights measures weight dispersion, not full survey-design precision. Weights have mean 1 except released weight2. Selection-IPW values were re-estimated using Panel A; “trimming” means capping, not deleting cases. Table S8 uses the capped, renormalized weights and fixed-weight HC0 inference. SD, standard deviation; P99, 99th percentile; IPW, inverse-probability weighting.

# **Supplementary Table S5. Survey weighting and sample-selection diagnostics (continued)**

## **Panel C. Contribution of each interview mode to the combined weight**

| **Interview mode** | **N** | **Sample share (%)** | **Mean combined weight** | **Share of total combined weight (%)** | **Maximum combined weight** |
| --- | --- | --- | --- | --- | --- |
| Face-to-face | 5,261 | 94.2 | 0.556 | 52.4 | 1.172 |
| Telephone | 324 | 5.8 | 8.213 | 47.6 | 18.464 |

Note. Combined weights have mean 1 and are untrimmed. Weight shares are within-mode weight sums divided by the total.

## **Panel D. Category distributions by interview mode and weighting specification**

| **Joint sport-engagement category** | **Face-to-face, unweighted (%)** | **Telephone, unweighted (%)** | **Full sample, combined weights (%)** | **Face-to-face, household weights (%)** |
| --- | --- | --- | --- | --- |
| Low engagement | 55.5 | 21.9 | 41.3 | 55.1 |
| Active-only engagement | 24.1 | 17.3 | 18.4 | 21.0 |
| Spectator-only engagement | 10.5 | 26.2 | 18.4 | 13.0 |
| Dual engagement | 9.8 | 34.6 | 21.9 | 11.0 |

Note. Column percentages use face-to-face n = 5,261, telephone n = 324 or full-sample N = 5,585. Household-weighted estimates use weight1. Rounding may affect totals.

**Supplementary Table S6. Sensitivity analyses of exercise, live attendance and their joint categories**

Note. N = 5,585; n is unweighted. Contrasts: education score unit, doubling of (income + 1), status rung, or urban versus rural. Only Panel B includes province indicators; other primary covariates remain. Estimation details: Panel F. OR, odds ratio; RRR, relative risk ratio; CI, confidence interval.

**Panel A. Joint categories with attendance required at least several times a month**

| **Outcome / frequency** | **n** | **Predictor** | **Unweighted RRR (95% CI)** | **p** | **Combined-weight RRR (95% CI)** | **p** |
| --- | --- | --- | --- | --- | --- | --- |
| Active-only engagement | 1,752 | Education | 1.119 (1.098–1.141) | <0.001 | 1.154 (1.106–1.205) | <0.001 |
|  |  | Personal income | 0.980 (0.969–0.992) | 0.001 | 0.983 (0.957–1.011) | 0.229 |
|  |  | Subjective social status | 1.052 (1.017–1.088) | 0.003 | 1.033 (0.952–1.121) | 0.436 |
|  |  | Urban vs. rural | 1.573 (1.349–1.835) | <0.001 | 1.227 (0.874–1.722) | 0.237 |
| Spectator-only engagement | 131 | Education | 1.055 (1.001–1.113) | 0.047 | 1.013 (0.923–1.113) | 0.782 |
|  |  | Personal income | 1.005 (0.970–1.041) | 0.800 | 1.013 (0.931–1.102) | 0.765 |
|  |  | Subjective social status | 1.048 (0.947–1.159) | 0.363 | 1.238 (1.034–1.482) | 0.020 |
|  |  | Urban vs. rural | 1.161 (0.745–1.809) | 0.509 | 1.048 (0.419–2.618) | 0.920 |
| Dual engagement | 204 | Education | 1.113 (1.063–1.165) | <0.001 | 1.102 (1.009–1.204) | 0.031 |
|  |  | Personal income | 0.983 (0.958–1.009) | 0.204 | 0.927 (0.877–0.980) | 0.007 |
|  |  | Subjective social status | 1.079 (0.996–1.169) | 0.062 | 1.007 (0.856–1.185) | 0.931 |
|  |  | Urban vs. rural | 1.656 (1.116–2.456) | 0.012 | 1.275 (0.544–2.992) | 0.576 |

Note. Reference: below both thresholds (n = 3,498). Frequent exercise remains daily or several times a week. Lower-frequency attendance is reclassified, not excluded. Panel F provides the matched comparator.

| **Binary outcome** | **n** | **Predictor** | **Unweighted OR (95% CI)** | **p** | **Combined-weight OR (95% CI)** | **p** |
| --- | --- | --- | --- | --- | --- | --- |
| Frequent exercise | 1,956 | Education | 1.110 (1.090–1.131) | <0.001 | 1.145 (1.100–1.190) | <0.001 |
|  |  | Personal income | 0.981 (0.970–0.993) | 0.001 | 0.972 (0.946–0.999) | 0.043 |
|  |  | Subjective social status | 1.056 (1.022–1.091) | 0.001 | 1.005 (0.933–1.083) | 0.887 |
|  |  | Urban vs. rural | 1.544 (1.319–1.808) | <0.001 | 1.324 (0.927–1.890) | 0.122 |
| Any live attendance | 1,267 | Education | 1.084 (1.060–1.110) | <0.001 | 1.075 (1.021–1.132) | 0.006 |
|  |  | Personal income | 1.018 (1.003–1.032) | 0.018 | 1.020 (0.988–1.053) | 0.219 |
|  |  | Subjective social status | 1.072 (1.032–1.114) | <0.001 | 1.060 (0.969–1.159) | 0.205 |
|  |  | Urban vs. rural | 1.630 (1.354–1.962) | <0.001 | 1.114 (0.751–1.651) | 0.592 |

**Panel B. Separate binary logistic regressions**

Note. References: exercise below the frequent threshold (n = 3,629) and no attendance (n = 4,318). Pooled province indicators are retained.

**Supplementary Table S6 (continued)**

**Panel C1. Original five-category exercise item**

Reference: never exercise (n = 2,296).

| **Outcome / frequency** | **n** | **Predictor** | **Unweighted RRR (95% CI)** | **p** | **Combined-weight RRR (95% CI)** | **p** |
| --- | --- | --- | --- | --- | --- | --- |
| Several times a year or less | 690 | Education | 1.089 (1.060–1.120) | <0.001 | 1.097 (1.030–1.168) | 0.004 |
|  |  | Personal income | 1.006 (0.988–1.025) | 0.510 | 1.018 (0.979–1.059) | 0.363 |
|  |  | Subjective social status | 1.124 (1.070–1.181) | <0.001 | 1.186 (1.060–1.326) | 0.003 |
|  |  | Urban vs. rural | 2.347 (1.883–2.925) | <0.001 | 1.781 (1.053–3.011) | 0.031 |
| Several times a month | 643 | Education | 1.148 (1.112–1.186) | <0.001 | 1.232 (1.151–1.318) | <0.001 |
|  |  | Personal income | 1.024 (1.003–1.044) | 0.022 | 1.034 (0.988–1.083) | 0.146 |
|  |  | Subjective social status | 1.103 (1.046–1.163) | <0.001 | 1.123 (0.991–1.273) | 0.068 |
|  |  | Urban vs. rural | 2.480 (1.944–3.164) | <0.001 | 2.550 (1.587–4.098) | <0.001 |
| Several times a week | 792 | Education | 1.182 (1.149–1.216) | <0.001 | 1.248 (1.169–1.333) | <0.001 |
|  |  | Personal income | 0.989 (0.973–1.006) | 0.202 | 0.992 (0.953–1.032) | 0.688 |
|  |  | Subjective social status | 1.127 (1.073–1.184) | <0.001 | 1.124 (1.010–1.251) | 0.033 |
|  |  | Urban vs. rural | 2.241 (1.793–2.800) | <0.001 | 2.150 (1.292–3.579) | 0.003 |
| Daily | 1,164 | Education | 1.156 (1.129–1.184) | <0.001 | 1.233 (1.172–1.297) | <0.001 |
|  |  | Personal income | 0.983 (0.968–0.997) | 0.021 | 0.972 (0.938–1.008) | 0.127 |
|  |  | Subjective social status | 1.092 (1.047–1.138) | <0.001 | 1.108 (1.018–1.206) | 0.018 |
|  |  | Urban vs. rural | 2.110 (1.748–2.547) | <0.001 | 1.438 (0.947–2.183) | 0.088 |

Note. Covariates match Panel C2. Main-text Table 3 reports these multinomial estimates without p-values.

**Supplementary Table S6 (continued)**

**Panel C2. Original five-category live-attendance item**

Reference: never attend (n = 4,318).

| **Outcome / frequency** | **n** | **Predictor** | **Unweighted RRR (95% CI)** | **p** | **Combined-weight RRR (95% CI)** | **p** |
| --- | --- | --- | --- | --- | --- | --- |
| Several times a year or less | 932 | Education | 1.092 (1.065–1.120) | <0.001 | 1.079 (1.022–1.138) | 0.006 |
|  |  | Personal income | 1.020 (1.004–1.036) | 0.015 | 1.036 (1.001–1.072) | 0.042 |
|  |  | Subjective social status | 1.061 (1.016–1.107) | 0.007 | 1.037 (0.936–1.148) | 0.490 |
|  |  | Urban vs. rural | 1.663 (1.368–2.021) | <0.001 | 1.048 (0.692–1.587) | 0.826 |
| Several times a month | 203 | Education | 1.047 (1.001–1.096) | 0.047 | 1.078 (0.978–1.188) | 0.130 |
|  |  | Personal income | 1.014 (0.988–1.041) | 0.298 | 0.983 (0.932–1.035) | 0.510 |
|  |  | Subjective social status | 1.035 (0.954–1.124) | 0.405 | 0.959 (0.804–1.144) | 0.642 |
|  |  | Urban vs. rural | 1.490 (1.031–2.153) | 0.034 | 1.489 (0.663–3.344) | 0.335 |
| Several times a week | 103 | Education | 1.065 (1.011–1.122) | 0.018 | 0.973 (0.889–1.064) | 0.546 |
|  |  | Personal income | 0.981 (0.946–1.016) | 0.282 | 0.946 (0.869–1.029) | 0.197 |
|  |  | Subjective social status | 1.066 (0.955–1.189) | 0.253 | 1.203 (1.007–1.436) | 0.041 |
|  |  | Urban vs. rural | 1.285 (0.755–2.187) | 0.355 | 0.956 (0.336–2.716) | 0.932 |
| Daily | 29 | Education | 1.175 (1.042–1.326) | 0.009 | 1.225 (1.061–1.415) | 0.006 |
|  |  | Personal income | 1.021 (0.931–1.120) | 0.656 | 1.064 (0.946–1.197) | 0.299 |
|  |  | Subjective social status | 1.232 (1.040–1.460) | 0.016 | 1.576 (1.145–2.169) | 0.005 |
|  |  | Urban vs. rural | 0.789 (0.295–2.108) | 0.637 | 0.305 (0.054–1.730) | 0.180 |

Note. Daily attendance: n = 29. The lowest nonzero category combines single and repeated visits. Province indicators are omitted because of separation.

**Supplementary Table S6 (continued)**

**Panel D. Five joint categories after splitting the original low-engagement reference**

| **Outcome / frequency** | **n** | **Predictor** | **Unweighted RRR (95% CI)** | **p** | **Combined-weight RRR (95% CI)** | **p** |
| --- | --- | --- | --- | --- | --- | --- |
| Occasional exercise without attendance | 829 | Education | 1.113 (1.083–1.143) | <0.001 | 1.158 (1.090–1.229) | <0.001 |
|  |  | Personal income | 1.008 (0.991–1.025) | 0.380 | 1.014 (0.977–1.053) | 0.453 |
|  |  | Subjective social status | 1.070 (1.021–1.121) | 0.005 | 1.066 (0.940–1.208) | 0.318 |
|  |  | Urban vs. rural | 2.293 (1.858–2.829) | <0.001 | 2.055 (1.334–3.166) | 0.001 |
| Active-only engagement | 1,326 | Education | 1.146 (1.120–1.171) | <0.001 | 1.220 (1.161–1.283) | <0.001 |
|  |  | Personal income | 0.984 (0.970–0.997) | 0.020 | 0.996 (0.964–1.029) | 0.806 |
|  |  | Subjective social status | 1.087 (1.044–1.131) | <0.001 | 1.159 (1.070–1.255) | <0.001 |
|  |  | Urban vs. rural | 1.939 (1.618–2.324) | <0.001 | 1.423 (0.984–2.059) | 0.061 |
| Spectator-only engagement | 637 | Education | 1.110 (1.078–1.144) | <0.001 | 1.128 (1.057–1.204) | <0.001 |
|  |  | Personal income | 1.027 (1.006–1.047) | 0.010 | 1.054 (1.008–1.102) | 0.020 |
|  |  | Subjective social status | 1.110 (1.052–1.171) | <0.001 | 1.226 (1.111–1.354) | <0.001 |
|  |  | Urban vs. rural | 2.014 (1.594–2.545) | <0.001 | 1.214 (0.721–2.044) | 0.465 |
| Dual engagement | 630 | Education | 1.233 (1.194–1.273) | <0.001 | 1.254 (1.169–1.346) | <0.001 |
|  |  | Personal income | 0.994 (0.975–1.013) | 0.546 | 0.985 (0.942–1.030) | 0.507 |
|  |  | Subjective social status | 1.120 (1.061–1.182) | <0.001 | 1.085 (0.973–1.210) | 0.140 |
|  |  | Urban vs. rural | 2.804 (2.175–3.614) | <0.001 | 1.733 (0.974–3.083) | 0.061 |

Note. Reference: never exercise and never attend (n = 2,163). Occasional exercise means several times a month or several times a year or less, without attendance. Other categories are unchanged; Panel G compares their probability contrasts.

**Composition of the original low-engagement category (n = 2,992)**

| **Exercise frequency; all report no attendance** | **Unweighted n** | **Within-category %** | **Weighted within-category %** |
| --- | --- | --- | --- |
| Never exercise | 2,163 | 72.3 | 63.0 |
| Exercise several times a year or less | 452 | 15.1 | 18.6 |
| Exercise several times a month | 377 | 12.6 | 18.5 |

**Supplementary Table S6 (continued)**

**Panel E. Observed frequencies and category composition**

**E1. Cross-tabulation of the two original items (unweighted counts)**

| **Exercise frequency / live attendance** | **Never** | **Yearly or less** | **Monthly** | **Weekly** | **Daily** | **Total** |
| --- | --- | --- | --- | --- | --- | --- |
| Never | 2,163 | 103 | 13 | 13 | 4 | 2,296 |
| Several times a year or less | 452 | 209 | 25 | 4 | 0 | 690 |
| Several times a month | 377 | 194 | 46 | 22 | 4 | 643 |
| Several times a week | 497 | 205 | 57 | 28 | 5 | 792 |
| Daily | 829 | 221 | 62 | 36 | 16 | 1,164 |
| Total | 4,318 | 932 | 203 | 103 | 29 | 5,585 |

Note. Yearly or less, monthly and weekly abbreviate the corresponding “several times” categories. Exact frequencies are unavailable.

**E2. Category sizes under each joint classification**

| **Classification** | **Joint category** | **Unweighted n** | **Unweighted %** | **Weighted %** |
| --- | --- | --- | --- | --- |
| Primary four categories | Low engagement | 2,992 | 53.6 | 41.3 |
|  | Active-only engagement | 1,326 | 23.7 | 18.4 |
|  | Spectator-only engagement | 637 | 11.4 | 18.4 |
|  | Dual engagement | 630 | 11.3 | 21.9 |
| Stricter attendance | Below both frequency thresholds | 3,498 | 62.6 | 56.0 |
|  | Active-only engagement | 1,752 | 31.4 | 32.1 |
|  | Spectator-only engagement | 131 | 2.3 | 3.7 |
|  | Dual engagement | 204 | 3.7 | 8.2 |
| Split-reference five categories | Never exercise and never attend | 2,163 | 38.7 | 26.0 |
|  | Occasional exercise without attendance | 829 | 14.8 | 15.3 |
|  | Active-only engagement | 1,326 | 23.7 | 18.4 |
|  | Spectator-only engagement | 637 | 11.4 | 18.4 |
|  | Dual engagement | 630 | 11.3 | 21.9 |

Note. Weighted percentages in Table S6 use combined survey/item-observation weights. Rounding may affect classification and within-category totals.

**E3. Attendance several times a year or less within the primary attendance-containing categories**

| **Primary category** | **Yearly-or-less n / category n** | **Within-category %** | **Weighted within-category %** |
| --- | --- | --- | --- |
| Spectator-only engagement | 506 / 637 | 79.4 | 79.9 |
| Dual engagement | 426 / 630 | 67.6 | 62.7 |

Note. Percentages use each category’s own denominator. Single visits cannot be isolated.

**Supplementary Table S6 (continued)**

**Panel F. Four-category comparator with the same adjustment set as Panels A and D**

| **Outcome / frequency** | **n** | **Predictor** | **Unweighted RRR (95% CI)** | **p** | **Combined-weight RRR (95% CI)** | **p** |
| --- | --- | --- | --- | --- | --- | --- |
| Active-only engagement | 1,326 | Education | 1.110 (1.087–1.133) | <0.001 | 1.153 (1.102–1.205) | <0.001 |
|  |  | Personal income | 0.982 (0.970–0.995) | 0.007 | 0.990 (0.960–1.021) | 0.522 |
|  |  | Subjective social status | 1.062 (1.023–1.102) | 0.002 | 1.114 (1.017–1.221) | 0.021 |
|  |  | Urban vs. rural | 1.535 (1.295–1.819) | <0.001 | 1.130 (0.804–1.589) | 0.482 |
| Spectator-only engagement | 637 | Education | 1.071 (1.041–1.102) | <0.001 | 1.057 (0.995–1.123) | 0.070 |
|  |  | Personal income | 1.025 (1.005–1.044) | 0.012 | 1.046 (1.005–1.089) | 0.027 |
|  |  | Subjective social status | 1.081 (1.028–1.137) | 0.003 | 1.175 (1.050–1.315) | 0.005 |
|  |  | Urban vs. rural | 1.530 (1.226–1.911) | <0.001 | 0.926 (0.565–1.519) | 0.761 |
| Dual engagement | 630 | Education | 1.188 (1.152–1.225) | <0.001 | 1.174 (1.099–1.254) | <0.001 |
|  |  | Personal income | 0.992 (0.975–1.010) | 0.396 | 0.978 (0.937–1.021) | 0.311 |
|  |  | Subjective social status | 1.090 (1.035–1.147) | 0.001 | 1.038 (0.919–1.173) | 0.550 |
|  |  | Urban vs. rural | 2.135 (1.671–2.729) | <0.001 | 1.338 (0.770–2.324) | 0.302 |

Note. Reference: primary low engagement (n = 2,992). Province indicators are omitted; covariates and weights match Panels A and D.

**Adjustment sets and estimation**

All models include the four focal predictors, age centered at 50 and its square, sex, self-rated health, agricultural hukou, partnered status and survey mode. Panel B also includes province indicators, pooling the 12 provinces with fewer than 20 complete cases.

Province indicators are omitted because separation persists after pooling: Panel A, code 22/spectator-only; Panel D, code 12/occasional exercise; Panel C2, eight empty cells. Panels C1 and F use matching adjustment sets.

Combined weights equal weight2 / p_m (Table S5), with mean 1 and no trimming. Weighted models maximize the weighted log likelihood. Two-sided Wald inference uses HC0 with fixed weights, excluding clustering, stratification and weight-estimation uncertainty. P-values are unadjusted.

All 14 models converged with finite coefficients; all 5,585 respondents were retained.

**Supplementary Table S6 (continued)**

**Panel G. Probability-scale comparison of the unchanged joint categories**

**G1. Unweighted models**

| **Predictor contrast** | **Unchanged outcome** | **Four-category model Change, pp (95% CI)** | **Split-reference model Change, pp (95% CI)** | **Difference (split − four), pp** |
| --- | --- | --- | --- | --- |
| Education: 6 to 12 years-equivalent | Active-only | 7.65 (5.58 to 9.71) | 7.58 (5.42 to 9.74) | -0.07 |
|  | Spectator-only | 1.05 (-0.53 to 2.62) | 0.86 (-0.80 to 2.51) | -0.19 |
|  | Dual | 6.36 (5.11 to 7.60) | 6.44 (5.15 to 7.72) | +0.08 |
| Income: 3,000 to 50,000 yuan | Active-only | -1.40 (-2.24 to -0.56) | -1.46 (-2.29 to -0.62) | -0.06 |
|  | Spectator-only | 1.20 (0.47 to 1.94) | 1.19 (0.45 to 1.92) | -0.02 |
|  | Dual | -0.26 (-0.88 to 0.37) | -0.28 (-0.90 to 0.35) | -0.02 |
| Subjective status: rung 3 to rung 6 | Active-only | 1.78 (-0.09 to 3.64) | 1.81 (-0.06 to 3.67) | +0.03 |
|  | Spectator-only | 1.32 (-0.08 to 2.72) | 1.33 (-0.06 to 2.73) | +0.01 |
|  | Dual | 1.41 (0.07 to 2.75) | 1.43 (0.09 to 2.77) | +0.02 |
| Residence: rural to urban | Active-only | 4.42 (1.57 to 7.28) | 4.11 (1.25 to 6.97) | -0.32 |
|  | Spectator-only | 1.76 (-0.29 to 3.81) | 1.60 (-0.46 to 3.65) | -0.16 |
|  | Dual | 4.80 (2.82 to 6.79) | 4.64 (2.65 to 6.62) | -0.17 |

**G2. Combined-weight models**

| **Predictor contrast** | **Unchanged outcome** | **Four-category model Change, pp (95% CI)** | **Split-reference model Change, pp (95% CI)** | **Difference (split − four), pp** |
| --- | --- | --- | --- | --- |
| Education: 6 to 12 years-equivalent | Active-only | 7.91 (4.75 to 11.07) | 7.99 (4.57 to 11.40) | +0.08 |
|  | Spectator-only | -1.26 (-6.19 to 3.68) | -1.78 (-7.02 to 3.46) | -0.53 |
|  | Dual | 8.91 (4.90 to 12.92) | 9.00 (4.74 to 13.27) | +0.10 |
| Income: 3,000 to 50,000 yuan | Active-only | -0.78 (-2.55 to 1.00) | -0.81 (-2.59 to 0.98) | -0.03 |
|  | Spectator-only | 3.05 (0.88 to 5.22) | 3.07 (0.90 to 5.24) | +0.02 |
|  | Dual | -2.02 (-4.38 to 0.34) | -2.04 (-4.40 to 0.31) | -0.02 |
| Subjective status: rung 3 to rung 6 | Active-only | 2.77 (-0.94 to 6.47) | 2.97 (-0.74 to 6.69) | +0.21 |
|  | Spectator-only | 5.12 (1.06 to 9.18) | 5.28 (1.21 to 9.35) | +0.16 |
|  | Dual | -1.96 (-6.62 to 2.70) | -1.74 (-6.40 to 2.93) | +0.22 |
| Residence: rural to urban | Active-only | 0.92 (-3.89 to 5.72) | 0.34 (-4.50 to 5.19) | -0.57 |
|  | Spectator-only | -2.79 (-9.79 to 4.20) | -3.19 (-10.19 to 3.80) | -0.40 |
|  | Dual | 4.08 (-3.55 to 11.71) | 3.54 (-4.12 to 11.19) | -0.54 |

Note. Higher-minus-lower contrasts retain other covariates at observed values. Predictions are averaged equally in G1 and with fixed combined weights in G2; both models omit province. CIs use the delta method and HC0. The last column is the descriptive difference between point estimates before rounding. pp, percentage points.

**Supplementary Table S7. Alternative specifications of socioeconomic indicators**

**Panel A1. Categorical education: unweighted adjusted probabilities, % (95% CI)**

| **Educational attainment (assigned score)** | **n** | **Low engagement** | **Active-only engagement** | **Spectator-only engagement** | **Dual engagement** |
| --- | --- | --- | --- | --- | --- |
| No schooling or literacy classes (0) | 584 | 73.3 (68.6–77.5) | 13.6 (10.9–16.9) | 10.4 (7.3–14.7) | 2.7 (1.4–5.1) |
| Primary (6) | 1,251 | 63.2 (60.2–66.1) | 21.0 (18.7–23.5) | 10.0 (8.1–12.3) | 5.8 (4.4–7.6) |
| Junior middle (9) | 1,589 | 54.0 (51.6–56.4) | 24.9 (22.9–27.0) | 11.9 (10.4–13.7) | 9.2 (7.8–10.7) |
| Upper-secondary or vocational (12) | 1,027 | 46.3 (43.3–49.3) | 27.7 (25.0–30.5) | 11.8 (10.1–13.8) | 14.2 (12.3–16.3) |
| College (15) | 486 | 41.7 (37.0–46.6) | 27.1 (22.9–31.7) | 13.1 (10.7–15.9) | 18.1 (15.0–21.7) |
| Bachelor’s (16) | 570 | 38.7 (33.9–43.7) | 32.2 (27.5–37.4) | 12.2 (9.9–15.0) | 16.8 (13.8–20.4) |
| Postgraduate (19) | 78 | 34.5 (23.5–47.5) | 38.5 (27.3–51.0) | 10.8 (6.2–18.0) | 16.2 (10.1–25.0) |

**Panel A2. Categorical education: combined-weight-model adjusted probabilities, % (95% CI)**

| **Educational attainment (assigned score)** | **n** | **Low engagement** | **Active-only engagement** | **Spectator-only engagement** | **Dual engagement** |
| --- | --- | --- | --- | --- | --- |
| No schooling or literacy classes (0) | 584 | 75.6 (66.3–82.9) | 13.9 (10.2–18.8) | 9.6 (4.8–18.1) | 0.9 (0.3–3.3) |
| Primary (6) | 1,251 | 63.6 (57.8–69.0) | 21.2 (17.6–25.2) | 9.1 (5.8–14.1) | 6.2 (3.6–10.3) |
| Junior middle (9) | 1,589 | 52.8 (48.6–57.0) | 22.0 (19.0–25.3) | 13.7 (10.8–17.2) | 11.4 (8.6–15.1) |
| Upper-secondary or vocational (12) | 1,027 | 44.9 (38.5–51.5) | 30.5 (25.0–36.8) | 12.2 (8.8–16.7) | 12.3 (8.9–16.8) |
| College (15) | 486 | 41.6 (32.1–51.8) | 24.6 (16.9–34.4) | 14.2 (9.6–20.4) | 19.6 (13.4–27.8) |
| Bachelor’s (16) | 570 | 35.6 (26.5–45.9) | 41.4 (31.4–52.2) | 9.8 (6.2–15.1) | 13.2 (8.2–20.5) |
| Postgraduate (19) | 78 | 16.5 (6.3–37.0) | 58.6 (36.4–77.8) | 6.4 (2.2–17.3) | 18.4 (8.1–36.6) |

Note. Both models use N = 5,585, low engagement as reference, and primary covariates/province pooling. Education uses six indicators (reference: score 0), without interactions; n is the unweighted group count.

Predictions are averaged equally over all 5,585 covariate profiles; A2 applies combined weights during fitting only. Pointwise CIs use HC0 and logit-transformed delta-method limits, with weights and covariates fixed. Rounding may affect totals.

**Supplementary Table S7. Alternative specifications of socioeconomic indicators (continued)**

**Panel A3. Robust joint Wald tests of the linear education-score restrictions**

| **Model** | **N** | **Wald χ²** | **df** | **p** |
| --- | --- | --- | --- | --- |
| Unweighted | 5,585 | 13.87 | 15 | 0.536 |
| Combined survey/item-observation weight | 5,585 | 23.95 | 15 | 0.066 |

Note. Joint Wald tests impose β(s) = (s/6)β(6) for s = 9, 12, 15, 16 and 19 in each non-reference equation (15 restrictions). P-values are unadjusted; non-rejection does not establish linearity. HC0 excludes clustering, stratification and weight-estimation uncertainty.

**Panel A4. Upper-end dual-engagement contrast: postgraduate minus bachelor’s education**

| **Categorical model** | **Difference, pp** | **95% CI, pp** | **p** |
| --- | --- | --- | --- |
| Unweighted | -0.63 | -8.27 to 7.01 | 0.872 |
| Combined survey/item-observation weight | +5.25 | -8.72 to 19.22 | 0.461 |

Note. Differences use the predictions and within-model joint covariance from A1–A2, with probability-scale delta-method CIs and no multiplicity adjustment. Bachelor’s n = 570; postgraduate n = 78. pp, percentage points.

## **Panel B. Personal income quartiles: adjusted predicted probabilities (%)**

| **Personal income quartile** | **Low engagement** | **Active-only engagement** | **Spectator-only engagement** | **Dual engagement** |
| --- | --- | --- | --- | --- |
| Quartile 1 | 54.6 | 25.9 | 8.6 | 10.9 |
| Quartile 2 | 54.7 | 24.7 | 10.1 | 10.5 |
| Quartile 3 | 51.4 | 24.6 | 13.1 | 10.9 |
| Quartile 4 | 54.2 | 20.8 | 12.9 | 12.1 |

## **Panel C. Subjective social status groups: adjusted predicted probabilities (%)**

| **Subjective social status** | **Low engagement** | **Active-only engagement** | **Spectator-only engagement** | **Dual engagement** |
| --- | --- | --- | --- | --- |
| Low (1–3) | 56.8 | 22.9 | 10.0 | 10.3 |
| Middle (4–6) | 52.6 | 24.0 | 11.9 | 11.5 |
| High (7–10) | 48.3 | 25.7 | 13.0 | 13.1 |

## **Panel D. Residential context in four categories: adjusted predicted probabilities (%)**

| **Residential context** | **Low engagement** | **Active-only engagement** | **Spectator-only engagement** | **Dual engagement** |
| --- | --- | --- | --- | --- |
| Rural village | 59.4 | 21.8 | 10.9 | 7.9 |
| Town | 48.5 | 22.7 | 15.9 | 12.9 |
| Urban fringe/transition zone | 50.6 | 25.4 | 10.3 | 13.7 |
| City center | 48.3 | 27.0 | 11.6 | 13.1 |

Note. Panels B–D give unweighted average predictions from alternative primary-model specifications, with other covariates at observed values. Rounding may affect totals.

**Supplementary Table S7. Alternative specifications of socioeconomic indicators (continued)**

## **Panel E. Income transformation comparison**

| **Transformation** | **Category** | **Original-scale contrast** | **Change in adjusted probability (pp)** | **Income-term coefficient** | **Coefficient p-value** |
| --- | --- | --- | --- | --- | --- |
| Log transformation after adding one yuan | Active-only engagement | 3,000 to 50,000 yuan | −1.32 | −0.0237 | 0.016 |
|  | Spectator-only engagement |  | 1.21 | 0.0381 | 0.010 |
|  | Dual engagement |  | −0.17 | −0.0067 | 0.633 |
| Inverse hyperbolic sine transformation | Active-only engagement | 3,000 to 50,000 yuan | −1.24 | −0.0223 | 0.016 |
|  | Spectator-only engagement |  | 1.13 | 0.0354 | 0.011 |
|  | Dual engagement |  | −0.17 | −0.0068 | 0.605 |

Note. Income is in yuan. Coefficients and p-values concern unit changes in transformed income; probability contrasts compare 50,000 minus 3,000 yuan (Q3−Q1). Both transformations retain zeros. Separate zero-income models are in Panel H. pp, percentage points.

## **Panel F. Influence of observed incomes above 1,000,000 yuan**

| **Income rule** | **N** | **Outcome versus low engagement** | **RRR** | **95% CI** | **p** |
| --- | --- | --- | --- | --- | --- |
| Primary unweighted | 5,585 | Active-only engagement | 0.977 | 0.958–0.996 | 0.016 |
|  |  | Spectator-only engagement | 1.039 | 1.009–1.069 | 0.010 |
|  |  | Dual engagement | 0.993 | 0.967–1.021 | 0.633 |
| Income ≤1,000,000 yuan | 5,577 | Active-only engagement | 0.976 | 0.957–0.995 | 0.015 |
|  |  | Spectator-only engagement | 1.037 | 1.008–1.068 | 0.013 |
|  |  | Dual engagement | 0.994 | 0.967–1.021 | 0.643 |

Note. Eight incomes above 1,000,000 yuan are excluded; zeros are retained. Reference: low engagement. Income RRRs are per unit increase in ln(income + 1), as in Table S4.

**Supplementary Table S7. Alternative specifications of socioeconomic indicators (continued)**

**Panel G. Distribution of zero personal income**

| **Sample** | **N** | **Zero income, n** | **Unweighted %** |
| --- | --- | --- | --- |
| Full analytical sample | 5,585 | 932 | 16.7 |
| Face-to-face | 5,261 | 899 | 17.1 |
| Telephone | 324 | 33 | 10.2 |

Note. Counts and percentages are unweighted. With combined weights, zero income accounts for 14.9%. Reported zeros are valid observed incomes.

**Panel H. Separate modeling of zero and positive personal incomes**

| **Weighting** | **Income contrast** | **Active-only engagement RRR (95% CI)** | **Spectator-only engagement RRR (95% CI)** | **Dual engagement RRR (95% CI)** |
| --- | --- | --- | --- | --- |
| Unweighted | Zero income vs. 20,000 yuan | 1.274 (1.048–1.549) | 0.759 (0.569–1.014) | 1.202 (0.905–1.596) |
| Unweighted | Doubling of positive income | 1.000 (0.952–1.050) | 1.107 (1.029–1.191) | 1.083 (1.010–1.160) |
| Combined weight | Zero income vs. 20,000 yuan | 1.242 (0.826–1.866) | 0.510 (0.270–0.964) | 1.606 (0.865–2.982) |
| Combined weight | Doubling of positive income | 1.078 (0.947–1.227) | 1.081 (0.924–1.265) | 1.199 (1.025–1.403) |

Note. Both models use N = 5,585, low engagement as reference, and primary covariates/province pooling. Joint income terms are a zero-income indicator and ln(income/20,000) for positive incomes, set to 0 otherwise. Each pair of rows comes from one model: zero versus 20,000 yuan (sample median), and a doubling of positive income.

Combined weights follow Table S5. Pointwise CIs use HC0 with fixed weights. Both models converged.

# **Supplementary Table S8. Sensitivity analyses of weighting, covariate adjustment and sample selection**

## **Panel A. Model estimates**

| **Model** | **N** | **Predictor** | **Active-only RRR** | **Spectator-only RRR** | **Dual engagement RRR** |
| --- | --- | --- | --- | --- | --- |
| Primary unweighted | 5,585 | Education | 1.104* | 1.072* | 1.185* |
|  |  | Income, per doubling of (income + 1) | 0.984* | 1.027* | 0.995 |
|  |  | Subjective social status | 1.066* | 1.095* | 1.100* |
|  |  | Urban residence | 1.465* | 1.500* | 2.287* |
| Combined survey/item-observation weight | 5,585 | Education | 1.147* | 1.062 | 1.190* |
|  |  | Income, per doubling of (income + 1) | 0.991 | 1.047* | 0.989 |
|  |  | Subjective social status | 1.119* | 1.194* | 1.037 |
|  |  | Urban residence | 1.092 | 0.851 | 1.412 |
| Face-to-face only, unweighted | 5,261 | Education | 1.102* | 1.077* | 1.187* |
|  |  | Income, per doubling of (income + 1) | 0.983* | 1.024* | 0.998 |
|  |  | Subjective social status | 1.064* | 1.077* | 1.130* |
|  |  | Urban residence | 1.500* | 1.609* | 2.429* |
| Face-to-face, household weight | 5,261 | Education | 1.120* | 1.083* | 1.209* |
|  |  | Income, per doubling of (income + 1) | 0.967* | 1.019 | 0.990 |
|  |  | Subjective social status | 1.095* | 1.067* | 1.115* |
|  |  | Urban residence | 1.392* | 1.525* | 1.984* |
| Selection-IPW combined weight (trimmed) | 5,585 | Education | 1.144* | 1.058 | 1.184* |
|  |  | Income, per doubling of (income + 1) | 0.992 | 1.046* | 0.987 |
|  |  | Subjective social status | 1.132* | 1.209* | 1.055 |
|  |  | Urban residence | 1.099 | 0.861 | 1.458 |

Note. RRR, relative risk ratio; *p < 0.05 using respondent-level HC0 standard errors, with weights fixed. Reference: low engagement. Contrasts: education score unit, status rung, doubling of (income + 1), or urban versus rural. Selection-IPW estimates were re-estimated using the inclusion model and capped weights in Table S5.

# **Supplementary Table S8. Sensitivity analyses of weighting, covariate adjustment and sample selection (continued)**

## **Panel A. Model estimates (continued)**

| **Model** | **N** | **Predictor** | **Active-only RRR** | **Spectator-only RRR** | **Dual engagement RRR** |
| --- | --- | --- | --- | --- | --- |
| Self-rated health omitted | 5,585 | Education | 1.106* | 1.073* | 1.189* |
|  |  | Income, per doubling of (income + 1) | 0.988 | 1.029* | 1.001 |
|  |  | Subjective social status | 1.086* | 1.106* | 1.131* |
|  |  | Urban residence | 1.502* | 1.515* | 2.346* |
| Household registration omitted | 5,585 | Education | 1.110* | 1.079* | 1.189* |
|  |  | Income, per doubling of (income + 1) | 0.985* | 1.027* | 0.996 |
|  |  | Subjective social status | 1.068* | 1.098* | 1.101* |
|  |  | Urban residence | 1.569* | 1.619* | 2.388* |
| Province indicators omitted | 5,585 | Education | 1.110* | 1.071* | 1.188* |
|  |  | Income, per doubling of (income + 1) | 0.982* | 1.025* | 0.992 |
|  |  | Subjective social status | 1.062* | 1.081* | 1.090* |
|  |  | Urban residence | 1.535* | 1.530* | 2.135* |

Note. *p < 0.05. Each unweighted model retains N = 5,585 and omits only the named covariate. Weight construction is in Table S5.

## **Panel B. Face-to-face-only unweighted model: 95% confidence intervals**

| **Predictor contrast** | **Active-only engagement RRR (95% CI)** | **Spectator-only engagement RRR (95% CI)** | **Dual engagement RRR (95% CI)** |
| --- | --- | --- | --- |
| Education, per years-equivalent unit | 1.102 (1.079–1.126) | 1.077 (1.044–1.112) | 1.187 (1.146–1.229) |
| Income, per doubling of (income + 1) | 0.983 (0.970–0.997) | 1.024 (1.003–1.046) | 0.998 (0.978–1.019) |
| Subjective social status, per rung | 1.064 (1.024–1.106) | 1.077 (1.021–1.136) | 1.130 (1.068–1.196) |
| Urban vs. rural residence | 1.500 (1.250–1.801) | 1.609 (1.258–2.059) | 2.429 (1.835–3.216) |

Note. N = 5,261; reference: low engagement. Primary covariates remain except constant survey mode. Original province pooling remains; empty levels are omitted and an observed province is the reference. CIs use HC0.

**Supplementary Table S8. Sensitivity analyses of weighting, covariate adjustment and sample selection (continued)**

**Panel C. Income associations with and without work-status adjustment in the same face-to-face sample**

| **Weighting** | **Income specification (reported contrast)** | **Work status adjusted** | **Active-only engagement RRR (95% CI)** | **Spectator-only engagement RRR (95% CI)** | **Dual engagement RRR (95% CI)** |
| --- | --- | --- | --- | --- | --- |
| Unweighted | ln(income + 1) [doubling of (income + 1)] | No | 0.983 (0.970–0.997) | 1.024 (1.003–1.046) | 0.998 (0.978–1.019) |
| Unweighted | ln(income + 1) [doubling of (income + 1)] | Yes | 0.986 (0.971–1.001) | 1.020 (0.996–1.044) | 0.993 (0.970–1.017) |
| Unweighted | Zero indicator + positive-income log term [doubling of positive income] | No | 0.994 (0.946–1.045) | 1.120 (1.036–1.211) | 1.091 (1.011–1.176) |
| Unweighted | Zero indicator + positive-income log term [doubling of positive income] | Yes | 0.989 (0.939–1.041) | 1.110 (1.024–1.203) | 1.076 (0.995–1.164) |
| Household weight | ln(income + 1) [doubling of (income + 1)] | No | 0.967 (0.952–0.983) | 1.018 (0.994–1.043) | 0.990 (0.966–1.014) |
| Household weight | ln(income + 1) [doubling of (income + 1)] | Yes | 0.971 (0.954–0.989) | 1.017 (0.991–1.044) | 0.990 (0.962–1.019) |
| Household weight | Zero indicator + positive-income log term [doubling of positive income] | No | 0.948 (0.895–1.005) | 1.073 (0.991–1.163) | 1.009 (0.921–1.106) |
| Household weight | Zero indicator + positive-income log term [doubling of positive income] | Yes | 0.943 (0.887–1.003) | 1.068 (0.984–1.160) | 1.000 (0.911–1.099) |

Note. All eight models use the same 5,256 face-to-face cases; five of 5,261 lacked classifiable work status. Groups are working/temporarily absent (reference; n = 2,500), retired/not working (n = 975), and otherwise not working (n = 1,781), coded as in Table S1. “No” retains primary covariates without work-status indicators.

Income contrasts are bracketed. Zero-income models use the two terms defined in Table S1; displayed RRRs describe positive-income slopes. Reference: low engagement. Primary covariates and province pooling remain, omitting constant survey mode and empty province levels.

Household weights use weight1, normalized to mean 1 within this sample. Pointwise CIs use HC0 with fixed weights. All eight models converged.

Work status refers to the previous week; income to 2022. Telephone item A42new was not combined with face-to-face items a53–a54.

# **Supplementary Table S9. Collinearity, model, and interaction diagnostics**

## **Panel A. Correlations among focal predictors**

| **Predictor** | **Education** | **Log-transformed personal income** | **Subjective social status** | **Urban residence** |
| --- | --- | --- | --- | --- |
| Education | 1.000 | 0.236 | 0.112 | 0.417 |
| Log-transformed personal income | 0.236 | 1.000 | 0.084 | 0.274 |
| Subjective social status | 0.112 | 0.084 | 1.000 | 0.125 |
| Urban residence | 0.417 | 0.274 | 0.125 | 1.000 |

## **Panel B. Variance-inflation factors**

| **Predictor** | **VIF** |
| --- | --- |
| Education | 1.97 |
| Log-transformed personal income | 1.26 |
| Subjective social status | 1.08 |
| Urban residence | 1.77 |

## **Panel C. Selected model convergence**

| **Model** | **N** | **Converged** |
| --- | --- | --- |
| Primary unweighted | 5,585 | Yes |
| Combined survey/item-observation weight | 5,585 | Yes |
| Face-to-face only, unweighted | 5,261 | Yes |
| Face-to-face, household weight | 5,261 | Yes |
| Income ≤1,000,000 yuan | 5,577 | Yes |
| Self-rated health omitted | 5,585 | Yes |
| Stricter attendance, no province (both weightings) | 5,585 | Yes |
| Selection-IPW combined weight (trimmed) | 5,585 | Yes |
| Household registration omitted | 5,585 | Yes |
| Province indicators omitted | 5,585 | Yes |
| Asinh income model | 5,585 | Yes |
| Four-category residence model | 5,585 | Yes |
| Original five-category exercise, no province (both weightings) | 5,585 | Yes |
| Original five-category attendance, no province (both weightings) | 5,585 | Yes |
| Split-reference five categories, no province (both weightings) | 5,585 | Yes |
| Matched four-category comparator, no province (both weightings) | 5,585 | Yes |

Note. Correlations are Pearson coefficients; VIFs and correlations use complete cases. “Both weightings” means unweighted and combined-weight fits. Adjustment sets: Table S6, Panel F. VIF, variance-inflation factor.

# **Supplementary Table S9. Collinearity, model, and interaction diagnostics (continued)**

## **Panel D. Distribution of primary-model predicted probabilities**

| **Category** | **Min** | **P1** | **Median** | **P99** | **Max** | **n with probability <0.01** | **n with probability >0.99** |
| --- | --- | --- | --- | --- | --- | --- | --- |
| Low engagement | 0.0233 | 0.0984 | 0.5423 | 0.9117 | 0.9642 | 0 | 0 |
| Active-only engagement | 0.0180 | 0.0473 | 0.2252 | 0.4824 | 0.5688 | 0 | 0 |
| Spectator-only engagement | 0.0039 | 0.0098 | 0.0849 | 0.3910 | 0.5363 | 64 | 0 |
| Dual engagement | 0.0013 | 0.0032 | 0.0795 | 0.4824 | 0.7352 | 336 | 0 |

Note. P1 and P99 are the 1st and 99th percentiles; probabilities are on the 0–1 scale.

## Panel E. Sparse province-by-category cells in the primary four-category outcome

| **Diagnostic** | **Value** |
| --- | --- |
| Zero province-by-category cells before pooling | 14 |
| Zero province-by-category cells after pooling | 0 |
| Provinces with fewer than 20 complete cases | 12 |
| Pooled province codes | 3, 8, 10, 14, 15, 19, 20, 21, 25, 26, 29, 31 |
| Primary-model maximum gradient component (original fit) | 0.0401 |
| Primary-model log likelihood (original fit) | −5725.32 |

Note. Pooling uses total complete-case counts per province and retains all 5,585 cases. Diagnostics concern the primary four-category outcome; alternative outcomes are in Table S6, Panel F. Gradient and log likelihood are from the original fit.

**Panel F1. Education associations by residence: unweighted linear-score model**

| **Outcome versus low engagement** | **Rural RRR (95% CI)** | **Urban RRR (95% CI)** | **Urban-to-rural RRR ratio (95% CI)** |
| --- | --- | --- | --- |
| Active-only engagement | 1.143 (1.108–1.179) | 1.075 (1.048–1.102) | 0.940 (0.907–0.975) |
| Spectator-only engagement | 1.092 (1.045–1.140) | 1.054 (1.017–1.093) | 0.966 (0.918–1.016) |
| Dual engagement | 1.317 (1.243–1.395) | 1.131 (1.092–1.171) | 0.859 (0.807–0.913) |

**Panel F2. Education associations by residence: combined-weight linear-score model**

| **Outcome versus low engagement** | **Rural RRR (95% CI)** | **Urban RRR (95% CI)** | **Urban-to-rural RRR ratio (95% CI)** |
| --- | --- | --- | --- |
| Active-only engagement | 1.150 (1.082–1.222) | 1.143 (1.078–1.212) | 0.994 (0.923–1.070) |
| Spectator-only engagement | 1.140 (1.050–1.237) | 1.027 (0.944–1.116) | 0.901 (0.811–1.000) |
| Dual engagement | 1.226 (1.120–1.343) | 1.169 (1.074–1.272) | 0.954 (0.855–1.064) |

Note. N = 5,585. Original seven-level score (0, 6, 9, 12, 15, 16, 19) × residence models retain primary covariates/province pooling. RRRs are per score unit; the last column exponentiates the interaction coefficient. F1 is unweighted; F2 uses combined weights; both use HC0.

**Supplementary Table S9. Collinearity, model, and interaction diagnostics (continued)**

**Panel G1. Original education-by-residence cells and separation diagnosis**

| **Educational attainment** | **Residence** | **Low** | **Active-only** | **Spectator-only** | **Dual** | **Total n** |
| --- | --- | --- | --- | --- | --- | --- |
| No schooling or literacy classes | Rural | 370 | 47 | 14 | 4 | 435 |
| No schooling or literacy classes | Urban | 102 | 29 | 13 | 5 | 149 |
| Primary | Rural | 633 | 154 | 56 | 26 | 869 |
| Primary | Urban | 220 | 110 | 24 | 28 | 382 |
| Junior middle | Rural | 511 | 166 | 87 | 34 | 798 |
| Junior middle | Urban | 366 | 237 | 87 | 101 | 791 |
| Upper-secondary or vocational | Rural | 136 | 72 | 23 | 38 | 269 |
| Upper-secondary or vocational | Urban | 294 | 232 | 109 | 123 | 758 |
| College | Rural | 38 | 19 | 16 | 18 | 91 |
| College | Urban | 129 | 97 | 74 | 95 | 395 |
| Bachelor’s | Rural | 21 | 17 | 13 | 22 | 73 |
| Bachelor’s | Urban | 152 | 124 | 105 | 116 | 497 |
| Postgraduate | Rural | 0 | 3 | 2 | 2 | 7 |
| Postgraduate | Urban | 20 | 19 | 14 | 18 | 71 |

Note. Seven rural postgraduates include no low-engagement cases. Increasing their three non-reference logits together increases the likelihood without a finite maximum. The seven-level categorical interaction therefore has separation; ordinary Wald results are not reported.

Note. For this interaction only, bachelor’s and postgraduate levels are pooled: rural n = 80 and urban n = 568, with all outcomes represented. All 5,585 respondents are retained. Other levels, the primary analysis and Table S7 retain their original coding.

**Diagnostic evidence for the unrestricted seven-level model**

| **Model** | **Score tolerance** | **Maximum \|coefficient\|** | **Hessian condition number** | **Statistical status** |
| --- | --- | --- | --- | --- |
| Unweighted | 1e-06 | 11.14 | 2.01e+06 | No finite maximum |
| Unweighted | 1e-10 | 21.15 | 4.43e+10 | No finite maximum |
| Combined-weight | 1e-06 | 11.49 | 6.13e+06 | No finite maximum |
| Combined-weight | 1e-10 | 20.49 | 4.97e+10 | No finite maximum |

Note. Coefficients increase as the stopping tolerance tightens. The reproducible-code archive contains the likelihood-direction certificate and computational diagnostics.

**Supplementary Table S9. Collinearity, model, and interaction diagnostics (continued)**

**Panel G2. Omnibus interaction and linear-score specification tests**

| **No-interaction test: specification** | **Weighting** | **Wald χ²** | **df** | **p** |
| --- | --- | --- | --- | --- |
| Original seven-level score × residence | Unweighted | 29.16 | 3 | <0.001 |
| Original seven-level score × residence | Combined-weight | 4.11 | 3 | 0.250 |
| Matched six-level score × residence | Unweighted | 27.29 | 3 | <0.001 |
| Matched six-level score × residence | Combined-weight | 3.16 | 3 | 0.368 |
| Six-level categorical education × residence | Unweighted | 44.69 | 15 | <0.001 |
| Six-level categorical education × residence | Combined-weight | 8.66 | 15 | 0.895 |
| **Specification test within six-level categorical model** | **Weighting** | **Wald χ²** | **df** | **p** |
| Joint linear-score restrictions in both residence groups | Unweighted | 25.76 | 24 | 0.366 |
| Joint linear-score restrictions in both residence groups | Combined-weight | 28.42 | 24 | 0.243 |

Note. All models use the same 5,585 cases, outcome, covariates and province pooling. The matched score assigns 16 to bachelor’s or higher; scores 0, 6, 9, 12 and 15 remain. The 15-df Wald test sets five interaction coefficients to zero in each non-reference equation. The 24-df test imposes β(s) = (s/6)β(6), s = 9, 12, 15, 16, for education main and interaction effects in each equation.

Note. Tests use HC0. Additional p-values are unadjusted and outside the original nine-test Benjamini–Hochberg family (main-text Table 4). The 24-df test concerns the matched six-level categorical model.

**Panel G3. Numerical diagnostics for the six finite interaction models**

| **Specification** | **Weighting** | **Parameters** | **Iterations** | **Log likelihood** | **Max. \|score\|/N** | **Max. \|β\|** |
| --- | --- | --- | --- | --- | --- | --- |
| Original seven-level score | Unweighted | 96 | 7 | -5709.28 | 1.24e-16 | 6.65 |
| Matched six-level score | Unweighted | 96 | 7 | -5709.00 | 2.32e-16 | 6.70 |
| Six-level categorical | Unweighted | 120 | 7 | -5696.01 | 1.06e-12 | 6.03 |
| Original seven-level score | Combined-weight | 96 | 7 | -6048.77 | 2.25e-16 | 5.60 |
| Matched six-level score | Combined-weight | 96 | 7 | -6060.43 | 2.74e-16 | 5.64 |
| Six-level categorical | Combined-weight | 120 | 7 | -6010.72 | 8.66e-12 | 6.34 |

Note. All six models converged with finite coefficients and positive-definite information matrices. Log likelihoods are numerical diagnostics. Combined weights have mean 1 and are untrimmed. Inference excludes clustering, stratification and weight-estimation uncertainty.

**Supplementary Table S9. Collinearity, model, and interaction diagnostics (continued)**

**Panel H1. Six-level categorical interaction: unweighted probabilities and residence differences**

| **Joint outcome** | **Educational attainment** | **Rural probability, % (95% CI)** | **Urban probability, % (95% CI)** | **Urban minus rural, pp (95% CI)** |
| --- | --- | --- | --- | --- |
| Low engagement | No schooling or literacy classes | 80.0 (75.0–84.2) | 64.2 (55.4–72.1) | -15.85 (-25.29 to -6.42) |
|  | Primary | 69.4 (65.9–72.8) | 57.4 (52.5–62.3) | -11.99 (-17.82 to -6.17) |
|  | Junior middle | 61.4 (57.7–64.9) | 47.8 (44.4–51.3) | -13.51 (-18.69 to -8.34) |
|  | Upper-secondary or vocational | 50.2 (44.4–56.1) | 42.1 (38.5–45.7) | -8.19 (-15.27 to -1.11) |
|  | College | 37.7 (28.3–48.1) | 38.9 (33.7–44.3) | +1.22 (-10.02 to 12.46) |
|  | Bachelor’s or higher | 25.7 (17.2–36.5) | 35.7 (30.9–40.7) | +9.99 (-0.44 to 20.41) |
| Active-only engagement | No schooling or literacy classes | 11.1 (8.3–14.6) | 17.2 (12.3–23.7) | +6.17 (-0.20 to 12.54) |
|  | Primary | 17.7 (15.1–20.6) | 25.0 (21.2–29.3) | +7.34 (2.52 to 12.15) |
|  | Junior middle | 22.8 (19.8–26.2) | 26.7 (23.8–29.7) | +3.84 (-0.63 to 8.30) |
|  | Upper-secondary or vocational | 28.2 (23.1–34.0) | 28.4 (25.2–31.9) | +0.20 (-6.41 to 6.81) |
|  | College | 30.2 (20.9–41.6) | 27.3 (22.7–32.4) | -2.98 (-14.49 to 8.53) |
|  | Bachelor’s or higher | 40.9 (29.4–53.6) | 33.1 (28.3–38.4) | -7.81 (-20.55 to 4.94) |
| Spectator-only engagement | No schooling or literacy classes | 7.5 (4.6–12.2) | 14.1 (8.5–22.5) | +6.58 (-1.19 to 14.35) |
|  | Primary | 9.4 (7.2–12.1) | 9.3 (6.4–13.2) | -0.12 (-4.12 to 3.89) |
|  | Junior middle | 11.4 (9.3–14.0) | 12.1 (10.0–14.6) | +0.67 (-2.62 to 3.95) |
|  | Upper-secondary or vocational | 8.3 (5.6–12.0) | 13.4 (11.2–16.0) | +5.17 (1.17 to 9.18) |
|  | College | 14.0 (8.8–21.6) | 13.2 (10.5–16.5) | -0.81 (-7.76 to 6.14) |
|  | Bachelor’s or higher | 11.5 (6.9–18.7) | 12.5 (10.0–15.5) | +1.03 (-5.21 to 7.27) |
| Dual engagement | No schooling or literacy classes | 1.4 (0.5–3.7) | 4.5 (1.8–10.6) | +3.11 (-1.07 to 7.28) |
|  | Primary | 3.5 (2.3–5.2) | 8.3 (5.8–11.7) | +4.77 (1.62 to 7.93) |
|  | Junior middle | 4.4 (3.1–6.1) | 13.4 (11.2–16.0) | +9.01 (6.13 to 11.89) |
|  | Upper-secondary or vocational | 13.3 (9.9–17.6) | 16.1 (13.6–19.0) | +2.82 (-2.00 to 7.64) |
|  | College | 18.0 (11.6–26.9) | 20.6 (16.8–25.1) | +2.57 (-6.00 to 11.14) |
|  | Bachelor’s or higher | 21.9 (14.5–31.7) | 18.7 (15.1–22.8) | -3.21 (-12.29 to 5.87) |

Note. N = 5,585. Models include categorical education, residence, their interaction and primary covariates/province indicators. Predictions set education and residence jointly and are averaged equally over the same 5,585 other-covariate profiles in both models. The highest group pools bachelor’s and postgraduate levels (G1).

Pointwise probability CIs use logit-transformed delta-method limits; difference CIs use the full joint covariance on the probability scale. HC0 treats weights and covariates as fixed; no multiplicity adjustment is applied. Positive differences mean higher urban probabilities. Figure 4 plots H1–H2 differences. pp, percentage points.

**Supplementary Table S9. Collinearity, model, and interaction diagnostics (continued)**

**Panel H2. Six-level categorical interaction: combined-weight probabilities and residence differences**

| **Joint outcome** | **Educational attainment** | **Rural probability, % (95% CI)** | **Urban probability, % (95% CI)** | **Urban minus rural, pp (95% CI)** |
| --- | --- | --- | --- | --- |
| Low engagement | No schooling or literacy classes | 74.9 (67.2–81.3) | 75.2 (56.1–87.8) | +0.29 (-16.76 to 17.34) |
|  | Primary | 64.7 (57.6–71.2) | 62.5 (52.0–72.0) | -2.14 (-13.99 to 9.72) |
|  | Junior middle | 56.1 (49.2–62.7) | 49.9 (42.8–57.0) | -6.16 (-17.33 to 5.01) |
|  | Upper-secondary or vocational | 49.1 (42.2–56.0) | 43.2 (34.9–51.9) | -5.87 (-17.31 to 5.57) |
|  | College | 34.3 (24.3–45.9) | 41.8 (31.1–53.5) | +7.57 (-7.43 to 22.58) |
|  | Bachelor’s or higher | 21.2 (10.0–39.4) | 33.3 (24.5–43.5) | +12.18 (-4.45 to 28.81) |
| Active-only engagement | No schooling or literacy classes | 12.2 (8.3–17.6) | 16.5 (10.7–24.8) | +4.32 (-3.45 to 12.09) |
|  | Primary | 19.7 (15.5–24.8) | 22.4 (17.5–28.1) | +2.64 (-3.76 to 9.04) |
|  | Junior middle | 20.6 (16.7–25.2) | 22.9 (18.7–27.6) | +2.25 (-3.87 to 8.37) |
|  | Upper-secondary or vocational | 29.2 (23.1–36.3) | 31.4 (24.0–39.8) | +2.13 (-8.59 to 12.84) |
|  | College | 25.4 (16.1–37.7) | 24.6 (15.9–36.1) | -0.79 (-14.92 to 13.34) |
|  | Bachelor’s or higher | 48.9 (24.7–73.6) | 43.5 (33.6–54.0) | -5.40 (-33.69 to 22.90) |
| Spectator-only engagement | No schooling or literacy classes | 11.8 (6.8–19.9) | 7.4 (1.6–28.5) | -4.42 (-17.15 to 8.31) |
|  | Primary | 10.1 (6.1–16.5) | 8.6 (3.7–18.8) | -1.53 (-9.90 to 6.84) |
|  | Junior middle | 14.2 (9.9–20.0) | 14.1 (10.0–19.4) | -0.15 (-7.48 to 7.18) |
|  | Upper-secondary or vocational | 11.0 (6.7–17.4) | 11.7 (7.9–17.0) | +0.77 (-6.41 to 7.96) |
|  | College | 23.8 (11.9–41.9) | 12.1 (7.9–18.1) | -11.70 (-27.65 to 4.26) |
|  | Bachelor’s or higher | 15.9 (7.3–31.4) | 8.0 (5.0–12.7) | -7.90 (-20.13 to 4.33) |
| Dual engagement | No schooling or literacy classes | 1.0 (0.3–3.0) | 0.8 (0.1–6.7) | -0.19 (-2.27 to 1.90) |
|  | Primary | 5.4 (2.5–11.6) | 6.5 (3.3–12.4) | +1.03 (-5.14 to 7.20) |
|  | Junior middle | 9.1 (5.6–14.4) | 13.2 (8.6–19.7) | +4.07 (-3.59 to 11.72) |
|  | Upper-secondary or vocational | 10.7 (7.1–15.8) | 13.7 (9.2–20.0) | +2.98 (-4.10 to 10.05) |
|  | College | 16.5 (6.8–34.6) | 21.4 (14.2–30.9) | +4.91 (-10.44 to 20.25) |
|  | Bachelor’s or higher | 14.0 (4.7–35.2) | 15.1 (9.3–23.6) | +1.12 (-14.15 to 16.39) |

Note. N = 5,585; combined-weight fitting, with predictions averaged equally over the same covariate profiles as H1. Model specification, education grouping, standardization and CI methods follow H1.

**Supplementary Table S9. Collinearity, model, and interaction diagnostics (continued)**

**Panel H3. Change in the urban–rural probability difference from primary to bachelor’s-or-higher education**

| **Joint outcome** | **Model** | **Change, pp** | **95% CI, pp** | **p** |
| --- | --- | --- | --- | --- |
| Low engagement | Unweighted | +21.98 | 10.17 to 33.78 | <0.001 |
|  | Combined-weight | +14.32 | -6.28 to 34.92 | 0.173 |
| Active-only engagement | Unweighted | -15.14 | -28.66 to -1.63 | 0.028 |
|  | Combined-weight | -8.04 | -36.52 to 20.45 | 0.580 |
| Spectator-only engagement | Unweighted | +1.15 | -6.16 to 8.45 | 0.758 |
|  | Combined-weight | -6.37 | -21.03 to 8.29 | 0.394 |
| Dual engagement | Unweighted | -7.98 | -17.58 to 1.62 | 0.103 |
|  | Combined-weight | +0.09 | -16.07 to 16.24 | 0.992 |

Note. Contrast: (urban−rural) at bachelor’s or higher minus (urban−rural) at primary, using H1–H2 models. Negative values lower the signed gap, not necessarily its absolute size. CIs use the joint covariance of four standardized probabilities. These unadjusted two-level contrasts do not test monotonic narrowing or replace omnibus interaction tests.

Note. The reproducible analysis archive contains full coefficients, predictions, contrasts, separation diagnostics and independent validation. Individual-level data are not redistributed.
